# Incorporating buccal mass planar mechanics and anatomical features improves neuromechanical modeling of *Aplysia* feeding behavior

**DOI:** 10.1007/s00422-025-01017-1

**Published:** 2025-07-07

**Authors:** Michael J. Bennington, Ashlee S. Liao, Ravesh Sukhnandan, Bidisha Kundu, Stephen M. Rogers, Jeffrey P. Gill, Jeffrey M. McManus, Gregory P. Sutton, Hillel J. Chiel, Victoria A. Webster-Wood

**Affiliations:** 1 Department of Mechanical Engineering, Carnegie Mellon University, Pittsburgh, PA USA; 2 Department of Biomedical Engineering, Carnegie Mellon University, Pittsburgh, PA USA; 3 Robotics Institute, Carnegie Mellon University, Pittsburgh, PA USA; 4 McGowan Institute for Regenerative Medicine, Carnegie Mellon University, Pittsburgh, PA USA; 5 School of Life Sciences, University of Lincoln, Lincoln, LN6 7TS UK; 6 Population Health Sciences, Bristol Medical School, Clifton, Bristol, BS8 2BN UK; 7 Department of Biology, Case Western Reserve University, Cleveland, OH USA; 8 Department of Biomedical Engineering, Case Western Reserve University, Cleveland, OH USA; 9 Department of Neurosciences, Case Western Reserve University, Cleveland, OH USA

**Keywords:** *Aplysia californica*, Neuromechanics, Computational model, Quasistatic mechanics

## Abstract

To understand how behaviors arise in animals, it is necessary to investigate both the neural circuits and the biomechanics of the periphery. A tractable model system for studying multifunctional control is the feeding apparatus of the marine mollusk *Aplysia californica*. Previous *in silico* and *in roboto* models have investigated how the nervous and muscular systems interact in this system. However, these models are still limited in their ability to match *in vivo* data both qualitatively and quantitatively. We introduce a new neuromechanical model of *Aplysia* feeding that combines a modified version of a previously developed neural model with a novel biomechanical model that better reflects the anatomy and kinematics of *Aplysia* feeding. The model was calibrated using a combination of previously measured biomechanical parameters and hand-tuning to behavioral data. Using this model, simulated feeding experiments were conducted, and the resulting behavioral metrics were compared to animal data. The model successfully produces three key behaviors seen in *Aplysia* and demonstrates a good quantitative agreement with biting and swallowing behaviors. Additional work is needed to match rejection behavior quantitatively and to reflect qualitative observations related to the relative contributions of two key muscles, the hinge and I3. Future improvements will focus on incorporating the effects of deformable 3D structures in the simulated buccal mass.

**Supplementary Information:**

The online version contains supplementary material available at 10.1007/s00422-025-01017-1.

## Introduction

Animal behavior arises from the complex interactions between an animal’s nervous system, its muscles and their structural organization, and the environment (Chiel and Beer 1997; Full and Koditschek 1999). It remains an open question how these interactions produce behaviors; in addition, factors such as the size of the animal and the speed of movement profoundly shape the control challenges that brain and body must overcome (Sutton et al. 2023; Clemente and Dick 2023). In addition to enforcing constraints, the body provides critical feedback that regulates and modulates circuits in the nervous system (Bidaye et al. 2018; Merlet et al. 2021). Finally, the nervous system can exploit the architecture, compliance, and damping of biomechanical systems, which provide a morphological intelligence that reduces the required complexity of higher-level controllers (Valero-Cuevas and Santello 2017; Mo et al. 2020). These factors all combine to suggest that the nervous system cannot be studied in isolation (Chiel and Beer 1997; Krakauer et al. 2017), but rather in its appropriate mechanical and environmental context.

Brain-body interactions have been previously studied using neuromechanical models in many model organisms across phyla and in various behaviors. Walking and running are commonly studied, as they are vital to survival in most animals (e.g., fruit fly (Lobato-Rios et al. 2022), cockroach (Szczecinski et al. 2014), stick insect (Knops et al. 2013), rats (Deng et al. 2019), and humans (Song and Geyer 2015)). Other modeling studies have examined grasping and manipulation (Valero-Cuevas and Santello 2017), jumping (Cofer et al. 2010), and swimming (Tytell et al. 2010), amongst many other behaviors. In all of these systems, rigid skeletal components (e.g., bones and exoskeletons) provide the primary structure and constrain the system to a finite number of degrees of freedom. This allows modelers to take advantage of a host of well-established rigid body mechanics mathematical frameworks (e.g., Lagrangian mechanics, screw theory (Gal et al. 2003; Tsai and Yin 2019)) and computational tools (e.g., PyBullet (Coumans and Bai 2016−2019), Mujoco (Todorov et al. 2012), Animatlab (Cofer et al. 2010)). Additionally, the complex 3D geometries of muscles and other soft structures are often reduced to simple line elements, and their contact interactions neglected. This is often an acceptable simplification as the kinematics of these systems are predominantly dictated by the configuration of the rigid components and their articulations. As a consequence, the exact deformation of the muscles and soft elements may not be salient. These powerful modeling tools provide deep insights into the motor control of these endo- and exoskeletal systems. However, many animals lack significant rigid components. From nematodes and other worms to large cephalopods, animals across a large range of scales rely solely on soft tissues to locomote, feed, reproduce, and interact with the world, and even animals with rigid skeletons often contain soft-tissue motor systems (for example, the vertebrate tongue).

Studying movement in soft-bodied animals can be far more difficult than in animals with rigid skeletons and defined joints. Instead of the finite degrees of freedom associated with rigid body chains, soft-bodied systems have infinite degrees of freedom. A major challenge in modeling is to reduce this to a tractable dimensionality, through abstraction or simplifying assumptions. In addition, pronounced changes in the mechanical advantage and gross configuration of muscles caused by contact between multiple soft structural components must be dealt with. Not only are soft bodies a challenge to model, but often an even greater challenge to control. Despite these mechanical complexities, soft-bodied animals successfully perform numerous complex behaviors often with nervous systems comprised of relatively few neurons. How do the brain and body interact in these soft-bodied systems to create multifunctional behaviors in the face of their inherent mechanical complexity?

**Fig. 1:**
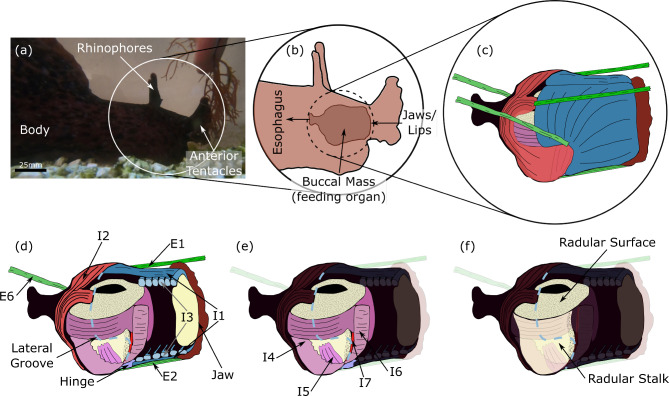
Overview of *Aplysia* feeding organ (buccal mass) anatomy. (**a**) An adult *Aplysia* feeding on *Gracilaria* seaweed. The white circle indicates the head of the animal, shown schematically in (**b**). The rhinophores and anterior tentacles both provide mechano- and chemosensory information to the animal. (**b**) Inside the head of the animal, the buccal mass connects anteriorly to the lips and posteriorly to the esophagus. Food enters through the lips and is carried through the buccal mass by the odontophore (grasper, (**e**)) where it is deposited into the esophagus. (**c**) False-colored anatomical drawing of the buccal mass musculature. All anatomical drawings ((**c**)-(**f**)) are modified with permission from Dai et al. (2022). The buccal mass is comprised of multiple interconnected muscles. (**d**) Cutaway anatomical drawing of the buccal mass showing internal structures. The outer muscles, which protract and retract the grasper, are shown labeled. Intrinsic muscles, which are wholly confined to the buccal mass, are designated “I” followed by a number and extrinsic muscles, which connect the buccal mass to the head, “E” followed by a number. (**e**) The internal musculature of the odontophore (grasper). (**f**) The grasping structure of the buccal mass is the radula (comprised of the radular stalk and radular surface), a tooth-covered cartilaginous structure articulated by the internal muscles of the odontophore

One tractable model with which to study the interplay between neural control and biomechanics in soft-bodied systems is the marine mollusk *Aplysia californica* (Fig. 1a) and its feeding organ, the buccal mass (Fig. 1b)(Webster-Wood et al. 2020; Sutton et al. 2004b; Novakovic et al. 2006; Dai et al. 2022; Evans et al. 1996; Gill and Chiel 2020; Liao et al. 2021; Lum et al. 2005; Hurwitz et al. 1996). *Aplysia* feeds on a variety of macroalgae (seaweed) (Kupfermann 1974), which it ingests as long strips of material, in contrast to the rasping from surfaces characteristic of most gastropods. The buccal mass is a fully soft system consisting of multiple interconnected muscular and cartilaginous structures (Howells 1942). The outer muscles of the buccal mass (Fig. 1(c-d)) form a tube-like structure that connects the lips of the animal to the esophagus. Within these outer muscles is a ball-like grasper, called the odontophore (Fig. 1e), whose muscles surround and articulate a toothed cartilaginous surface, called the radula (Fig. 1f). During feeding, the outer muscles of the buccal mass push the odontophore forward toward the lips (protraction) and backward toward the esophagus (retraction), while the muscles of the odontophore alternately open and close the radula on food (Howells 1942). Changing the phasing and relative magnitudes of these movements generates different feeding behaviors, the three best characterized of which are biting, swallowing, and rejection. Biting is an exploratory behavior, characterized by a strong protraction and weak retraction of the odontophore, where the animal attempts to grasp nearby food. Once the animal has successfully grasped food with its radula, it switches to a swallowing behavior, during which a strong retraction of the odontophore allows for food to be passed posteriorly to the esophagus. Finally, if the animal senses that what it has grasped is inedible, it can reverse the phasing of radular closing relative to biting and swallowing and instead push food out of the buccal mass in a rejection behavior (Kupfermann 1974; Neustadter et al. 2007).

The buccal mass neuromuscular system consists of a few key neurons that are large and identifiable that operate a limited number of muscles, which allows for behavior to be studied in a bottom-up fashion using *in vivo*, *in vitro*, *in silico*, and *in roboto* methods. Due to its slow behavioral cycling relative to its size, all feeding behaviors in the buccal mass remain quasi-static (Sutton et al. 2004b; Rogers et al. 2024), simplifying the analysis and modeling of the system’s mechanics since inertial forces can be neglected. This simplification applies across the entire size range of the animal (Rogers et al. 2024), spanning from around 150 mg post-metamorphosis to over 1 kg in adults (Audesirk 1979), meaning that the same model formulations can be used to model slugs of various sizes by making changes in model parameters.

Previous *in silico* and *in roboto* work has been dedicated to the modeling of *Aplysia*, at the component (Yu et al. 1999; Sutton et al. 2004a; Sukhnandan et al. 2024), subsystem (Novakovic et al. 2006; Sutton et al. 2004b; Costa et al. 2020), and system level (Mangan et al. 2005; Webster-Wood et al. 2020; Liao et al. 2021; Li et al. 2022; Dai et al. 2022; Li et al. 2024; Shaw et al. 2015). However, until now, models have either reduced the motion to single-axis translation (Novakovic et al. 2006; Sutton et al. 2004b; Webster-Wood et al. 2020; Li et al. 2022, 2024; Shaw et al. 2015), which ignored behaviorally relevant kinematics (Neustadter et al. 2007), or were limited in their quantitative comparisons with *in vivo* data (Mangan et al. 2005; Liao et al. 2021; Dai et al. 2022). *In roboto* and other physical models are appealing for investigating the buccal mass because, being fully soft-bodied, the buccal mass can be difficult to model in its entirety. Robotic models allow for multibody physics to be captured in physical models, greatly reducing computational and mathematical complexity (Koehl 2003; Gravish and Lauder 2018). However, *in roboto* models are limited by the available physical hardware and the accuracy of robotic components as analogs for the biological tissue. This is particularly true for soft robotic systems, which often must utilize bespoke actuator designs and fabrication modalities (El-Atab et al. 2020). While previous *in roboto* models of *Aplysia* (Mangan et al. 2005; Dai et al. 2022) have demonstrated the ability to produce multiple behaviors, there are still key discrepancies between their kinematics and those of the animal. *In silico* models, on the other hand, can model individual components to arbitrary precision, restricted predominantly by the availability of experimental data for calibration and computational resources. However, with increased complexity comes rapid increases in computational and calibration costs, so a balance must be struck between computational speed and the model’s fidelity to the biological system.

In this work, we propose a new system-level neuromechanical model of the feeding system of *Aplysia*. This model incorporates additional biomechanical features that were previously neglected (Sutton et al. 2004b; Novakovic et al. 2006; Webster-Wood et al. 2020; Mangan et al. 2005; Dai et al. 2022) with the aim of reducing the error between modeled behavior and that observed in the animal while maintaining computational efficiency. A previously described Boolean nervous system model (Webster-Wood et al. 2020; Dai et al. 2022) was modified to control a novel biomechanical model. This biomechanical model was developed using a demand-driven complexity framework, and thus, we added only the elements required to properly capture the animal’s behaviors. To this end, we hypothesized that the following simplifying assumptions (A) would still allow the model to adequately capture animal behaviors.

1. Due to the bilateral symmetry of the system, the relevant mechanics of the system project fully to the midsagittal plane, and therefore, only 2D geometry and mechanics are required.
2. The muscles and tissues of the system can be approximated using line element geometries.
3. Bulk tissue passive forces do not play a significant role other than what can be captured through the above-mentioned line element structures and can thus be neglected in the model.

In the model presented here, additional anatomical features, including both muscles and passive elastic elements, were introduced to replace previously abstracted mechanical units. All model parameters are either implemented directly from previous experiments or hand-tuned to match existing kinematics data (for full details, see Supporting Information Section 2). The simulations produced by this model were then quantitatively compared with animal data, and areas of modeling mismatch were identified. With this model, we can better understand the mechanics of the buccal mass and how it generates adaptive feeding behaviors in *Aplysia*, and more generally, determine control principles applicable to other soft-bodied animals and how they may differ from animals with rigid skeletons. At the same time, the most significant deviations of the model from the biological system will determine what new components or degrees of freedom must be added to future models.

## Materials and methods

### Modeling

#### Relevant animal anatomy

**Fig. 2:**
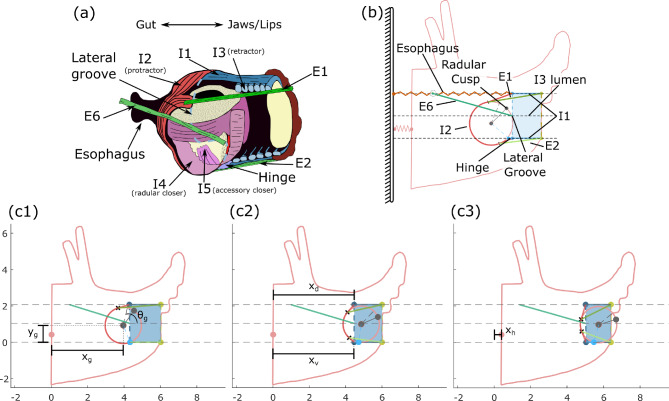
Comparison of (**a**) animal buccal musculature and (**b**) model anatomy. Analogous anatomical features share a color between (**a**) and (**b**). The outer intrinsic muscles (I1, I2, I3, and hinge) and the extrinsic muscles of the buccal mass are modelled using line element geometries. The odontophore is modelled as a rigid circle, with its musculature functionally abstracted as a single I4-like closer muscle. No grasper opener muscles were required for this model, and thus I7 was omitted. The rings of the I3 were modeled as an axial force acting at the I3 lumen midline but have no geometry to visualize. Note in (**b**) that the E6 attaches to the body wall at the same height as the esophagus, but the two structures do not interconnect. The point labelled as the “radular cusp” is where the model grabs food and represents the anterior-most point of the radular cleft (where food is grasped by the animal). In the model, we define the line from the radular cusp to the center of mass as the radular stalk axis. (**c**) Model degrees of freedom and multiple configurations. Frames (**c1**)–(**c3**) show three positions during the protraction of a biting behavior. Frame (**c3**) was slightly modified to ensure visibility of the $x_h$ degree of freedom. Positions are in normalized model units, with 1 model unit corresponding to the radius of the odontophore. Panel (a) was modified with permission from Dai et al. (2022)

Howells 1942 categorized the muscles of the buccal mass as either being intrinsic (I), entirely confined to the buccal mass, or extrinsic (E), connecting the buccal mass to the body wall. Muscles within the buccal mass can be roughly broken into two classes– the outer muscles that protract and retract the odontophore (Fig. 1d), and the muscles of the odontophore that articulate the radula (Fig. 1e) (Howells 1942). The anterior and posterior muscles interdigitate with each other and with the muscles of the odontophore at a region referred to as the lateral groove (shown as a light blue dashed line in Fig. 1(d-f)). The outer group of intrinsic muscles consists of the I1 and I3 muscles anteriorly and the I2 muscle posteriorly (Fig. 2d). The I1 is a thin muscle sheet that lies exterior and adheres tightly to the much larger underlying I3 muscle, which has muscle fiber orientations largely orthogonal to the I1. Together these muscles form a complex, with the I1 believed to be contributing to protraction (Howells 1942) and the I3 providing the primary retraction force (Lu et al. 2015). The I1/I3 is anchored anteriorly to the jaw (i.e. the most anterior region of the buccal mass that connects to the anterior body wall, Fig. 1d) (Howells 1942). The odontophore is moved through the lumen of the I1/I3 muscle complex during feeding behaviors.

The I2 muscle is another thin sheet that forms the back of the buccal mass, wrapping around the posterior of the odontophore and attaching both dorsally and ventrally to the I1/I3 complex at the lateral groove. The I2 serves as the primary protractor of the odontophore (Hurwitz et al. 1996).

Several extrinsic muscles help to position the buccal mass inside the head (Chiel et al. 1986). These extrinsic muscles are anatomically (though not necessarily physiologically) more similar to muscles in rigid body systems, as they are mostly 1-dimensional elements connecting two points inside the animal. The E1, E2, and E6 extrinsic muscles can be represented as acting mostly in the midsagittal plane (Fig. 1d) and are thus relevant for this model. The E1 muscle connects posteriorly to the dorsal arms of the I2 muscle and anteriorly near the jaw. The E6 muscle attaches to the lateral groove and projects posteriorly to the body wall. The E2 muscle connects the ventral edge of the lateral groove to the jaw, running mostly parallel to the ventral I1 muscle. The E1 and E6 muscles contract during protraction, and E2 during retraction (Jahan Parwar and Fredman 1983), though their contributions are not as critical to feeding effectiveness as the intrinsic muscles (Chiel et al. 1986).

The odontophore muscles that articulate the radula consist of the I4-I10 muscles (Fig. 1e, muscles I8-I10 not shown). The anatomy of these muscles and their functional roles in behavior are more complicated than the outer muscles that move the entire odontophore. In our model, the odontophore is assumed to be a rigid body (see “Model anatomy and degrees of freedom”), and thus the geometry of most of these muscles is not included. The functional role of the I4 muscles, which form much of the volume of the odontophore and act to close the radula on food (Morton and Chiel 1993), is included in the model, but no relevant geometry is included.

Finally, the hinge is an extended region of tissue formed by the interdigitation of the internal and external muscle, connecting the odontophore to the outer muscles of the buccal (Fig. 1d). The hinge connects the ventral edge of the lateral groove and has been hypothesized to assist in the retraction of the odontophore in biting and rejection (Sutton et al. 2004a).

#### Model anatomy and degrees of freedom

Key anatomical features of the musculature are included in our biomechanical model of the *Aplysia* buccal mass, which includes both intrinsic and extrinsic muscles (Fig. 2). From earlier models (Liao et al. 2021; Webster-Wood et al. 2020), the odontophore is modeled as a rigid circle and is acted upon by forces generated by the I2 protractor muscle (Hurwitz et al. 1996), the I3 retractor muscle (Neustadter et al. 2007; Church and Lloyd 1994), and hinge muscle (Sutton et al. 2004a). The I3 muscle is split into a bulk posterior portion that acts on the odontophore (Church and Lloyd 1994) and an anterior portion that generates a pinching force on food near the jaws (McManus et al. 2014). The I4 muscle acts internally to the rigid odontophore to generate forces on strips of food (Morton and Chiel 1993). In the model, the buccal mass sits inside a rigid head that is anchored to the global reference frame by a spring element representing the body wall of the animal.

To increase the anatomical accuracy of the model, the following features have been introduced: first, the I2 muscle was modeled as a chord that wraps conformally around the odontophore and attaches distally to a line (lateral groove) between the dorsal and ventral extents of the I3 lumen (Fig. 2b). Second, independent dorsal and ventral I1 muscles connect to the lateral groove and to the rigid head at the jaw. Third, in a previous model, the remaining muscles and connective tissue were previously represented as a single elastic element (Liao et al. 2021; Webster-Wood et al. 2020). To improve the biological fidelity of this model, this elastic element has been replaced by known muscles and tissues: a passive element representing the esophagus is attached between the dorsal extent of the lateral groove and the fixed reference frame; the E1, E2, and E6 extrinsic muscles were also added as active, contractile elements (Jahan Parwar and Fredman 1983; Chiel et al. 1986). E1 and E2 interdigitate with the I2 muscle and anchor at the jaw, whereas E6 attaches at the midpoint of the lateral groove and anchors posteriorly to the wall of the head. Additionally, the hinge muscle, previously modeled as acting at the odontophore center of mass (Webster-Wood et al. 2020), has been updated to better match the anatomy and has been modeled as attaching the ventrolateral odontophore to the ventral lateral groove. These anatomical features all capture some aspects of the true 3D geometry of the animal, but as part of our demand-driven complexity approach to this model, we hypothesize that the geometry of these muscular structures can be adequately captured using 1-dimensional line element geometries (A2).

To develop the equations of motion governing this system, the degrees of freedom (DoFs) must be defined. Though there are many distinct muscular and tissue elements in the system, many are geometrically constrained by one another. For this model, we further assume that due to the system’s bilateral symmetry, all relevant geometry and mechanics can be projected to the midsagittal plane of the head (A1). This removes two rotational DoFs and one translation DoF from each element. Finally, the head and the lateral groove are constrained to move only horizontally. Thus, six degrees of freedom remain (Fig. 2c)– the horizontal translation of the head ($x_h$), the horizontal translation of the dorsal and ventral extents of the lateral groove ($x_d$ and $x_v$, respectively), and the horizontal and vertical center-of-mass translation and the rotation of the odontophore ($x_g$, $y_g$, and $\theta _g$, respectively). Here, $\theta _g$ defines the angle from the horizontal axis to the odontophore midline (Fig. 2c1). A scleronomic constraint is added to account for passive reactive forces from the I3 lumen and the hinge that are not explicitly modeled, following assumption A3. Specifically, the odontophore is constrained by a pin-slot joint, where a pin rigidly attached to the odontophore is free to translate in the horizontal direction and rotate in the slot but cannot translate in the vertical direction. This couples the center-of-mass vertical translation $y_g$ and odontophore rotation $\theta _g$ by the kinematic constraint equation:

1 $$\begin{aligned} f(y_g,\theta _g) = y_g + R\sin {(\theta _g + \theta _H)} = 0 \end{aligned}$$

where $\theta _H$ is the fixed angle from the odontophore midline to the pin location on the odontophore, and *R* is the radius of the odontophore. In the animal, this coupling arises from complex interactions in a highly interdigitated structure. To fully model the interaction would require much more complicated mathematical methods that we deemed beyond the scope of our demand-driven complexity analysis. In addition to constraints imposed at the hinge, the system must be constrained to prevent interpenetration of the different muscular structures. Specifically, the odontophore must be prevented from passing through the ventral wall of the I3 lumen. In the animal, this arises naturally from these structures physically contacting each other; in the model, these constraints are captured by using rotational and translational inequality constraints (see “Penalty forces”).

**Fig. 3:**
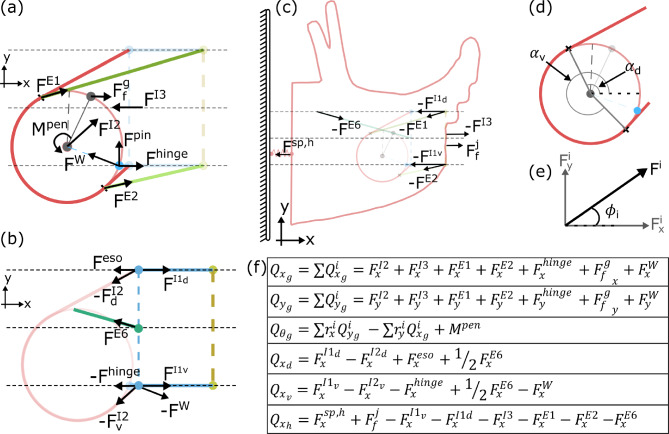
Summary of Model Mechanics. Forces exerted on the (**a**) odontophore, (**b**) dorsal and ventral lateral groove, and (**c**) head. Note, force arrows indicate the location of force application and *typical* direction of force application. All forces are defined as positive in the positive *x* and *y* directions, and the arrows drawn here do not indicate positive force direction. Arrow length also does not indicate force magnitude, as the magnitude will change throughout the feeding cycle. The force labeled $F^{pin}$ corresponds to the reaction force from the pin-slot joint equal to the Lagrange multiplier, $\lambda$. The olive dashed line indicates the jaw line (anterior edge of the I3 lumen) and where food is pinched by the anterior I3 muscle. (**d**) Definition of the tangency angles used to define the dorsal and ventral tangent points of the I2 muscle. (**e**) Definition of force decomposition and angle. (**f**) Summary of generalized forces associated with each coordinate. In the equation for the torques on the odontophore $Q_{\theta _g}$, $r_x^i$ and $r_y^i$ are the *x* and *y* components of the $i^{th}$ force’s moment arm relative to the odontophore center of mass. In the equation for forces $Q_{x_d}$ and $Q_{x_v}$, $F_x^{I2_i}$ is the portion of the I2 force vector associated with the angle $\alpha _i$ (see Eqn. 23). The value $M^{pen}$ is the proportional penalty torque associated with the inequality rotation constraint, and $F^{W}$ is the contact penalty force associated with the ventral wall of the I3 lumen. A list of symbols/variables and their description is provided in the Supporting Information Section 6

#### Quasi-static equations of motion

Using the degrees of freedom defined above, the governing model equations can be derived using a Lagrangian framework, where gravity is omitted, and all passive elastic forces are included with active muscle forces in the sum of generalized forces. This reduces the Lagrangian to the kinetic energy of the system:

2 $$\begin{aligned} L(q_i,{\dot{q}}_i) = \frac{1}{2}\sum _i M_i {\dot{q}}_i^2 \end{aligned}$$

where $M_i$ is the inertial parameter associated with the generalized coordinate $q_i$. The generalized coordinates correspond with the degrees of freedom introduced above. Next, it was assumed that there exist non-specific damping elements in the system that can be captured by the dissipative potential:

3 $$\begin{aligned} D(q_i) = \frac{1}{2}\sum _i c_i {\dot{q}}_i^2 \end{aligned}$$

where $c_i$ is the damping parameter associated with the $i^{th}$ generalized coordinate. These damping parameters collectively abstract the viscoelasticity of tissues and any drag effects caused by the system being submerged in fluid. The units of $M_i$ and $c_i$ will depend on whether the generalized coordinate *i* is a length/spatial coordinate or an angle.

The influence of the pin-slot constraint, $f(y_g,\theta _g)$ (Eq 1), can be incorporated using a Lagrange multiplier $\lambda$ representing a vertical reaction force on the odontophore pin ($F_{pin}$). Because the additional rotational and translational constraints are inequality constraints and will therefore not be enforced throughout the cycle, here they are weakly enforced using penalty methods (See “Penalty Forces” below). Finally, the generalized forces associated with the $i^{th}$ coordinate, accounting for both passive tissue and active muscle forces, are summarized as $Q_i$. The model equations of motion can then be derived from the Euler-Lagrange equations as:

4 $$\begin{aligned}& \frac{d}{dt} \frac{\partial L}{\partial {\dot{q}}_i} - \frac{\partial L}{\partial q_i} + \frac{\partial D}{\partial {\dot{q}}_i} + \lambda \frac{\partial f}{\partial q_i} = Q_{q_i}\\& \quad \implies M_i\ddot{q}_i + c_i{\dot{q}}_i + \lambda \frac{\partial f}{\partial q_i} = Q_{q_i} \end{aligned}$$

As previously observed, the inertia and accelerations in *Aplysia* feeding are several orders of magnitude smaller than other energetic sources and can thus be ignored (Sutton et al. 2004b; Webster-Wood et al. 2020; Liao et al. 2021; Kundu et al. 2022). This yields the general form:

5 $$\begin{aligned} {\dot{q}}_i = \frac{1}{c_{i}}\left( Q_{q_i} - \lambda \frac{\partial f}{\partial q_i} \right) \end{aligned}$$

For $x_h$, $x_v$, $x_d$, and $x_g$, $\partial f /\partial q_i = 0$, and the equation of motion (Eq 5) reduces to the scaled integration of the sum of forces. $y_g$ and $\theta _g$ are influenced by the constraint equation (Eq 1), with $\lambda$ specifically representing the vertical reaction force from the slot. The reaction force can be solved using the governing equation for $y_g$, specifically:

6 $$\begin{aligned} \lambda = \frac{(Q_{y_g} - c_{y_g}{\dot{y}}_g)}{\partial f / \partial y_g} = (Q_{y_g} - c_{y_g}{\dot{y}}_g) \end{aligned}$$

where ${\dot{y}}_g$ can be found by solving the constraint (Eq 1) for $y_g$ and differentiating with respect to time:

7 $$\begin{aligned} {\dot{y}}_g = -R\cos {(\theta _g + \theta _H)} \dot{\theta }_g \end{aligned}$$

Substituting this and the derivative of the constraint (Eq 1) with respect to $\theta _g$:

8 $$\begin{aligned} \partial f / \partial \theta _g = R\cos {(\theta _g + \theta _H)} \end{aligned}$$

into Eq 5 for $q_i = \theta _g$ and solving for $\dot{\theta }_g$ yields the governing equation:

9 $$\begin{aligned} \dot{\theta }_g = \frac{Q_{\theta _g} - Q_{y_g}(R\cos {(\theta _g + \theta _H)})}{c_{\theta _g} + c_{y_g}\left( R\cos {(\theta _g + \theta _H)}\right) ^2} \end{aligned}$$

The governing equation for $y_g$ then reduces to the derivative of the constraint equation (Eq 7). The components that sum together in $Q_i$ are determined by the active and passive muscle forces of the model (Fig. 3f). These components are

10 $$\begin{aligned} & \begin{aligned} Q_{x_g} = \sum _i Q_{x_g}^i = F_x^{I2} + F_x^{I3} +F_x^{E1} +&F_x^{E2} \\ +F_x^{hinge} + (F_f^{g})_x +&F_x^{W} \end{aligned} \end{aligned}$$

11 $$\begin{aligned} & \begin{aligned} Q_{y_g} = \sum _i Q_{y_g}^i = F_y^{I2} + F_y^{I3} +F_y^{E1} +&F_y^{E2} \\ + F_y^{hinge} + (F_f^{g})_y +&F_y^{W} \end{aligned} \end{aligned}$$

12 $$\begin{aligned} & Q_{\theta _g} = \sum _i r_x^i Q_{y_g}^i - \sum _i r_y^i Q_{x_g}^i + M^{pen} \end{aligned}$$

13 $$\begin{aligned} & Q_{x_d} = F_x^{I1_d} - F_x^{I2_d} + F^{eso} + \frac{1}{2}F_x^{E6} \end{aligned}$$

14 $$\begin{aligned} & Q_{x_v} = F_x^{I1_v} - F_x^{I2_v} - F_x^{hinge} + \frac{1}{2}F_x^{E6} - F_x^{W} \end{aligned}$$

15 $$\begin{aligned} & \begin{aligned} Q_{x_h} = F_x^{sp,h} + (F_f^j)_x - F_x^{I1_v} -&F_x^{I1_d} \\ - F_x^{I3} - F_x^{E1} - F_x^{E2} -&F_x^{E6} \end{aligned} \end{aligned}$$

#### Muscle and tissue tensions and forces

The generalized forces in the equations of motion consist of the active muscle and the passive tissue forces. All muscles and springs, except for the dorsal and ventral I1, generate tension with positive magnitudes (no load in compression), and the geometry of the system determines the 2D force vectors that result from these tensions. The passive elements parallel with the I1 muscles can generate reaction forces in compression, representing the bulk elasticity of the I1/I3 complex. For simplicity, this is wrapped into the I1 dorsal and ventral forces. Passive tension in the $j^{th}$ muscle/tissue is modeled as piecewise linear elastic elements governed by the equation:

16 $$\begin{aligned} T_p^j(L) = {\left\{ \begin{array}{ll} T^j_{max}\frac{L^j - L^j_0}{L^j_{max} - L^j_0} & L^j \ge L^j_0 \\ 0 & \text {otherwise} \end{array}\right. } \end{aligned}$$

where $L^j_0$ is the rest length of the $j^{th}$ element and $T^j_{max}$ (which has units of force) is the tension generated by the passive element at a length of $L^j_{max}$. Here, *j* indicates the element’s identifier and is not an exponent. For the passive elements parallel to the I1 muscles, this conditional is dropped, and the tension is simply:

17 $$\begin{aligned} T^{I1_k}(L) = T^{I1_k}_{max}\frac{L^{I1_k} - L^{I1_k}_0}{L^{I1_k}_{max} - L^{I1_k}_0} \end{aligned}$$

for $k\in [v,d]$. The active forces generated by the muscles of the system come from a simplified muscle model taken from Webster-Wood et al. (2020), where the active muscle tension *T* of the $j^{th}$ muscle is the first-order filtered response of activation *A*, which in turn is the first-order filtered response of neural input *N*. This double-first-order filter is described by the following system of equations:

18 $$\begin{aligned} T^j_a(t)= T^j_{max} {\tilde{T}}^j(t) \text { where } \end{aligned}$$

19 $$\begin{aligned} \frac{d{\tilde{T}}^j}{dt}= \frac{A^j(t) - {\tilde{T}}^j(t)}{\tau _j} \text { and} \end{aligned}$$

20 $$\begin{aligned} \frac{dA^j}{dt}= \frac{N^j(t) - A^j(t)}{\tau _j} \end{aligned}$$

Here, $A^j(t)$ and $N^j(t)$ are the normalized activation level $A\in [0,1]$ and the neural input to the $j^{th}$ muscle, and $\tau _j$ is the characteristic time constant associated with that muscle (Webster-Wood et al. 2020). For all muscles except for I2, this is a fixed value. As in Webster-Wood et al. (2020), the I2 has different time constants during activation and relaxation. The tension developed by the bulk posterior and anterior I3 muscle and the I4 muscle, comes only from this active component, as, in this model, there is no length associated with these muscles to generate passive forces. The tension in the I2, hinge, E1, E2, and E6 muscles are found as the sum of the active force model and the piecewise linear passive spring model:

21 $$\begin{aligned} T^j_{total}(t,L^j) = T^j_a(t) + T^j_p(L^j) \end{aligned}$$

The tension in the I1 line elements is the same, save for the passive element being the fully linear model, as mentioned for Eq 17.

The resulting force vectors from these muscle and tissue tensions can be found using the model geometry and constraints. First, the anterior I3 and I4 muscle forces only pinch food and, therefore, do not need to be decomposed into a 2D force vector. Next, the dorsal/ventral I1 and hinge muscles are constrained to the *x* direction, so the *x* component of their force vector equals the tension in the muscle, and the *y* component is 0. The E1, E2, and E6 muscles act as simple line elements, and their forces can be decomposed as:

22 $$\begin{aligned} {\bar{F}}^j(t,L^i) = T^j_{total}(t,L^j)\begin{bmatrix} \cos {(\phi _j)} \\ \sin {(\phi _j)} \end{bmatrix} \end{aligned}$$

where $\phi _j$ is the angle that the $j^{th}$ line element makes to the horizontal (Fig. 3e). This angle can be found knowing the fixed anchor and time-varying attachment points of the muscle. The attachment point for the E6 muscle is the lateral groove midpoint $( [x_d + x_v]/2, H_{lumen} / 2)$, where $H_{lumen}$ is the height of the I1/I3 lumen. The attachment points for the E1 and E2 muscles are the points on the dorsal and ventral edges of the odontophore where the I2 becomes tangent, respectively. These points are also used to calculate the net force generated by the wrapped I2 muscle. At each point along the circumference where the I2 muscle is in contact with the odontophore, an infinitesimal force vector points inwards along the radius. Integrating these force vectors from the dorsal to the ventral tangency point yields the net force vector:

23 $$\begin{aligned} {\bar{F}}^{I2}(t,L^{I2}) = T^{I2}_{total}(t,L^{I2})\begin{bmatrix} \sin {(\alpha _d)} - \sin {(\alpha _v)} \\ \cos {(\alpha _v)} - \cos {(\alpha _d)} \end{bmatrix} \end{aligned}$$

where $\alpha _d$ and $\alpha _v$ are the angles from the horizontal to the dorsal and ventral tangency points (Fig. 3d). These tangency points can be found from the odontophore center of mass location and the dorsal and ventral lateral groove points. The tangency point is defined by the point on the circle where the slope of the circle matches the slope of the line element running to the lateral groove anchor point. For the point $j\in [v,d]$, let

24 $$\begin{aligned} {\tilde{x}}_j= & x_j - x_{CoM} \end{aligned}$$

25 $$\begin{aligned} {\tilde{y}}_j= & y_j - y_{CoM} \end{aligned}$$

From these points, the tangency point $(x^j_T,y^j_T)$ can be found as:

26 $$\begin{aligned} x^j_{T}= & x_{CoM} + \min {\left( \frac{{\tilde{x}}_j R^2 \pm {\tilde{y}}_jR\sqrt{ {\tilde{x}}_j^2 + {\tilde{y}}_j^2 - R^2 }}{{\tilde{x}}_j^2 + {\tilde{y}}_j^2} \right) } \end{aligned}$$

27 $$\begin{aligned} y^j_T= & y_{CoM} + \frac{R^2 - {\tilde{x}}_j(x_T^j - x_{CoM})}{{\tilde{y}}_j} \end{aligned}$$

Using the $\min ()$ function ensures that the solution to the quadratic problem corresponds to the point on the posterior edge of the odontophore.

Next, the hinge force vector only points along the x-axis, so the y-component of the vector is always 0. To reflect the observation that, at low stretches, the hinge is unable to generate force (Sutton et al. 2004a), the hinge tension is multiplied by a piecewise linear mechanical advantage function $MA_{hinge}$. This function takes the form:

28 $$\begin{aligned} MA_{hinge}& =\text {sign}\left[ x_v - (x_g + R\cos {(\theta _g + \theta _H)})\right] \\ & \quad \times \min {\left( \frac{1}{0.5}\frac{L^{hinge} - L_0^{hinge}}{L_{max}^{hinge} - L_0^{hinge}}, 1\right) } \end{aligned}$$

The sign() function term determines the direction of force application based on whether the odontophore hinge point is in front of or behind the lateral groove. The second term scales the tension linearly while the hinge length is less than 50% of its maximum length, after which point it scales the tension uniformly.

Finally, the force from the bulk I3 muscle is found as the tension in the bulk I3 muscle multiplied by a piecewise linear mechanical advantage function. This function reflects the fact that I3 applies force to the odontophore through a contact pressure and is thus related to the contact area between the odontophore and I3. This piecewise linear mechanical advantage ($MA_{I3}(\delta )$) equation yields a maximal force when the odontophore is fully internal to the I3 lumen and zero force when the odontophore is fully outside of the I3 lumen:

29 $$\begin{aligned} MA_{I3}(\delta ) = {\left\{ \begin{array}{ll} -1 & \delta> 2R \\ -\delta / (2R) & \delta \in [0,2R] \\ 0 & \delta < 0 \end{array}\right. } \end{aligned}$$

where

30 $$\begin{aligned} \delta = x_g + R - 0.5(x_d + x_v) \end{aligned}$$

is the level of overlap between the odontophore and the I3 lumen. For the model presented here, the I3 force always acts along the horizontal lumen midline.

#### Penalty forces

As mentioned above, two inequality constraints (one translational and one rotational) were introduced to represent the contact forces coming from the I3 lumen which act to prevent the interpenetration of different structures. Because these constraints deal with inequalities (i.e., they are only enforced for particular configurations of the system), they cannot be handled directly using a Lagrange multiplier, as can the equality (pin-slot) constraint. Instead, these constraints are weakly enforced using linear penalty methods, where, if the governing inequality is not satisfied, a linear restoring force (or torque) is applied to the system in the direction that would act to enforce the constraint. For the rotational constraint, a torque is applied to the center of mass of the odontophore, but no reaction torque is applied to the I3 lumen as it has no rotational degree of freedom. The penalty torque is calculated as:

31 $$\begin{aligned} M^{pen} = {\left\{ \begin{array}{ll} k_{pen}\Delta \theta & \Delta \theta \ge 0 \\ 0 & \text {otherwise} \end{array}\right. } \end{aligned}$$

where $\Delta \theta =\left( \frac{3\pi }{2} - (\theta _g + \theta _h ) \right)$ and $k_{pen}$ is the stiffness of the penalty. The translational penalty force is applied if the point at $x_v$ enters into the odontophore while the odontophore is outside of the I3 lumen. To check if the odontophore is inside the I3 lumen, we check if $x_g> x_{hinge}$, which will only occur when the odontophore rotates into the I3 lumen. Here, $x_{hinge}$ is the x position of the hinge point on the odontophore. To check if the point at $x_v$ is inside the odontophore, we calculate the vector from the point at $x_v$ to the center of mass of the odontophore. Let that vector be

32 $$\begin{aligned} {\bar{l}}_v = \begin{bmatrix} x_g - x_v \\ y_g \end{bmatrix} \end{aligned}$$

The force is applied if $|{\bar{l}}_v| < R$. Therefore, the full penalty force is calculated as

33 $$\begin{aligned} {\bar{F}}^{W} = {\left\{ \begin{array}{ll} k_{W}\sqrt{(R^2 -|{\bar{l}}_v|^2)}\left( \frac{{\bar{l}}_V}{|{\bar{l}}_v|} \right) & \begin{aligned} x_g< & x_{hinge} \text { and } \\ &|{\bar{l}}_v| < R \end{aligned} \\ 0 & \text {otherwise} \end{array}\right. } \end{aligned}$$

where $k_{W}$ is the stiffness of the constraint.

#### Frictional forces between the odontophore and seaweed

The model buccal mass can exert forces on seaweed using both its grasper and its jaws. In both cases, the force is applied through frictional contact. For simplicity, the velocity dependence of this frictional force (van Geffen 2009) is ignored, and only static and Coulomb friction are considered, following the model presented in Webster-Wood et al. (2020). Briefly, for both the grasper (*g*) and the jaws (*j*), the frictional force ($F_f^g$ and $F_f^j$ respectively) is dependent on the state of slip. If the sum of other forces on the body in question is greater than the slip force, then the frictional force is determined by the grasping pressure and the coefficient of kinetic friction. The forces on the grasper and the jaws are, respectively, determined by the following:

34 $$\begin{aligned} & \sum F^g = F^{I2}_x + F^{I3}_x + F^{hinge}_x + F^{E1}_x + F^{E2}_x \end{aligned}$$

35 $$\begin{aligned} & \begin{aligned} \sum F^j =&F^{sp,h}_x - F^{I1_v}_x - F^{I1_d}_x - F^{I3}_x \\ &- F^{E1}_x - F^{E2}_x - F^{E6}_x \end{aligned} \end{aligned}$$

Note the force values $F^i_x$ can be positive or negative depending on the current configuration of the grasper. The penalty force $F^{W}$ is omitted from this calculation as it only contributes during forward motion (when the force on food is 0). When the constraint is met, this force will be zero, and the inclusion of the force only impacts the calculation through the introduction of numerical noise. Most muscle forces acting on the head are subtracted because they are defined relative to the grasper and lateral groove and thus react negatively on the head. In addition to the forces on the body, the grasping pressure must also be determined individually for the grasper and the jaws. For the grasper, this grasping pressure $P^g$ is equal to $F^{I4}$, and for the jaws, the pressure $P^{j}$ is equal to $F^{I3_{anterior}}$ (the I4 muscle is internal to the odontophore (Fig. 2a) and is consequently not shown in Fig. 3).

If the sum of the forces is not greater than the slip force, then the force is equal and opposite to the other applied forces. For both grasper and jaws, this frictional force only acts in the horizontal direction, assuming that the anchor of the seaweed is infinitely far away. Because friction only acts in the horizontal direction, only forces in the horizontal direction are considered in the slip evaluation. Additionally, the seaweed is assumed to support no force in compression, so force from friction can never be negative. For the grasper (*g*) and the jaws (*j*), the slip condition becomes:

36 $$\begin{aligned} \text {slip}_\beta = \bigg|\sum F^\beta \bigg|\ge \mu ^\beta _s P^\beta \end{aligned}$$

for $\beta \in [g,h]$. Together, with Eqs 34 and 35, the resulting friction force becomes:

37 $$\begin{aligned} F_f^\beta = {\left\{ \begin{array}{ll} \mu ^\beta _k P^\beta & \text {slip}_\beta ==1 \text { and } \sum F^\beta< 0 \\ -\sum F^\beta & \text {slip}_\beta ==0 \text { and } \sum F^\beta < 0 \\ 0 & \sum F^\beta> 0 \end{array}\right. } \end{aligned}$$

These forces are only applied if the seaweed is specified to be fixed (i.e. tethered and pulled taut). If the seaweed is specified to be detached, the frictional force is zero. For the case of no fixation, a secondary slip condition is tested for the grasper. Specifically, if $P^g> 0.5T^{I4}_{max}$, then the food is considered grasped ($\text {slip}_g = 0$). This indicator variable is used to calculate ingested/egested seaweed length (See “Model observables”). The jaw friction force is applied to the head, and the grasper friction force is applied to the grasper at a point defined by a fixed angle from the radular stalk axis, $\theta _f$. This point models the location of the cusp of the radular surface (Fig. 2) and is calculated as:

38 $$\begin{aligned} \begin{bmatrix} x_f \\ y_f \end{bmatrix} = \begin{bmatrix} x_g + R\cos (\theta _g - \theta _f) \\ y_g + R\sin (\theta _g - \theta _f) \end{bmatrix} \end{aligned}$$

#### Neural controller

**Fig. 4:**
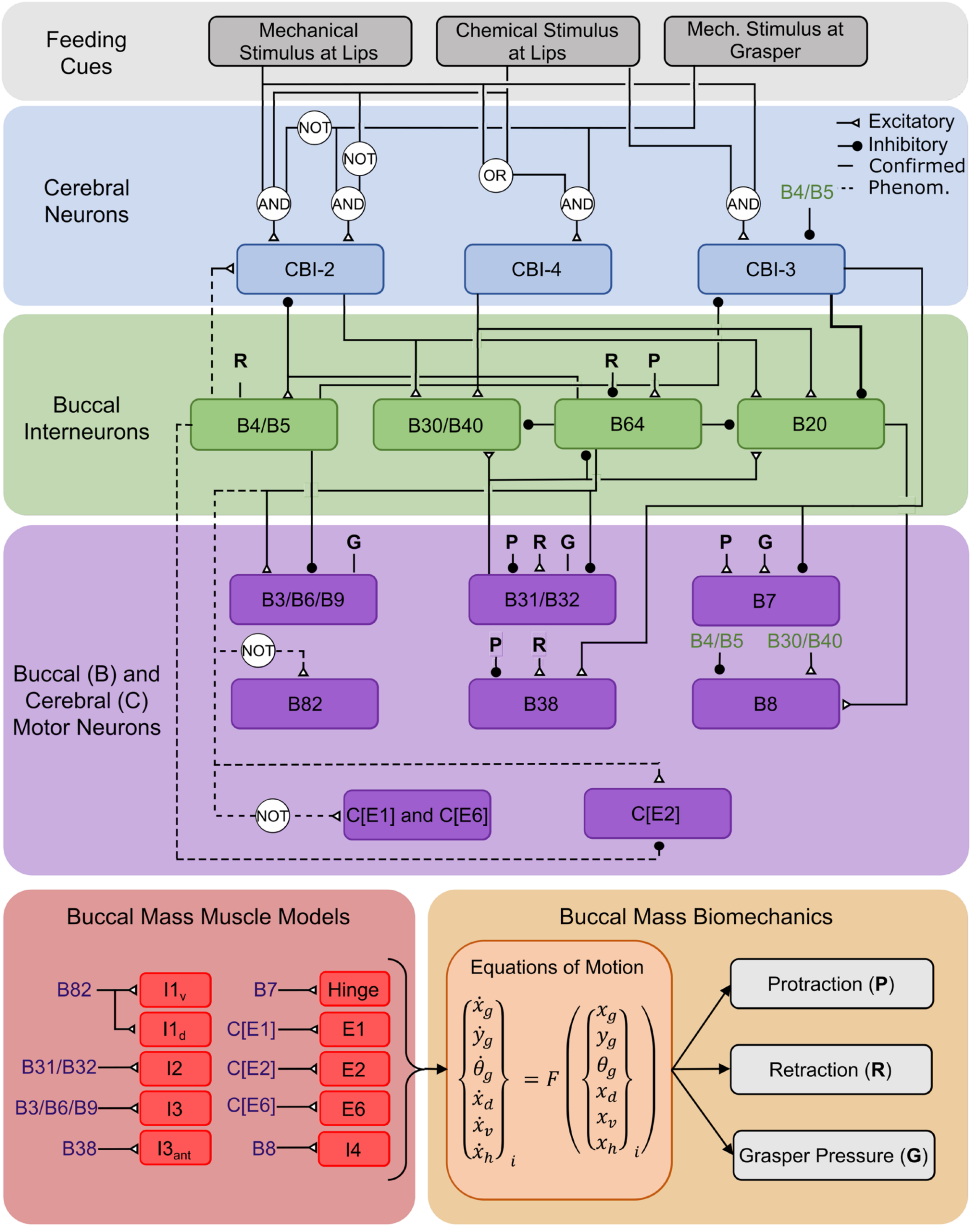
Neuromechanics Model Schematic. The neural circuitry of the buccal mass is modeled with a multilayered Boolean neural network modified from Webster-Wood et al. (2020). **Note:** for the sake of clarity, only key connections to the inter- and motor neurons are shown here. For a full accounting of the logic implemented for these neurons, we refer to Supporting Information Section 5 and Webster-Wood et al. (2020). Here, each neuron is represented by a 1 or 0, with 1 indicating activity and 0 indicating quiescence. The user specifies feeding cues, and the cerebral buccal interneurons (CBIs) integrate these feeding cues to determine the behavior to be performed. The buccal interneurons then combine CBI and proprioceptive signals to determine the phasing of behaviors. Finally, buccal motor neurons combine interneuron and proprioceptive information to activate muscles in the biomechanical models. These muscles generate forces that influence the equations of motion (See “Quasi-static equations of motion”). Kinematic and kinetic variables are then calculated from this mechanical model to serve as proprioceptive signals. Dashed lines represent phenomenological connections within the network and may not represent true pathways. The bold line from CBI-3 represents an overriding inhibition that negates any other excitatory signals. As the motor neurons for the included extrinsic muscles have not been identified, but may reside in the cerebral ganglion with other extrinsic motor neurons (Jahan Parwar and Fredman 1983), the motor neurons for extrinsic muscle Ex is labeled as C[Ex]

The neural controller utilized in this model is adapted from Webster-Wood et al. (2020) and Dai et al. (2022) (Fig. 4). For details of the model derivation and equations, we refer an interested reader to Webster-Wood et al. (2020). The complete set of equations and any modifications made for this model are provided in Supporting Information Section 5. Briefly, in the neural model, the activity of neurons is captured with Boolean variables, with 1 indicating spiking and 0 quiescence. The controller consists of three interconnected layers of neurons. Cerebral ganglion neurons process sensory cues to determine the behavior to be performed. Buccal ganglia interneurons then process cerebral and proprioceptive signals to determine the relative phasing of the model. Finally, buccal motor neurons process signals from the interneurons and cerebral neurons to activate the muscles in the biomechanical model. At each time step, the state of a given neuron is calculated using Boolean arithmetic operating on the states of presynaptic neurons from the previous time point. The neurons themselves are not dynamical in nature, but their behavior varies with time due to the feedback received from the biomechanical model, acting akin to a finite state machine.

All neurons included in Webster-Wood et al. (2020) are again included here, with additional neurons included for the muscles that were not present in previous models. All additional connections introduced in this model are phenomenological and may not reflect true connections. In Dai et al. (2022), the B43/B45 motor pool was included to activate the I1 muscles, with all I1 actuators sharing the same innervation. This motor pool was activated in retraction for all behaviors through connections to the CBI-3 cerebral neuron and the B64 interneuron. These connections were based on the assumption that I1 worked in tandem with the rest of the I1/I3 complex. However, closer examination of previous literature suggests that I1 and I3 have different functional roles. Church and Lloyd (1994) described I1 motor neurons as causing “jaw shortening,” in contrast to I3 motor neurons causing “jaw closure.” Howells, in the original 1942 description of *Aplysia*’s feeding system (Howells 1942), reported that I1 acts in conjunction with I2 and E1 to protract the odontophore. There is also evidence that the I1 motor neuron B45 may be doubly identified as B82 and that B82 can fire during the protraction phase (McManus 2014). Based on these findings, we hypothesize that I1 is activated during the protraction phase of the behavior and chose to model this protraction activity. The I1 motor neuron B43 is most active at the end of retraction after the cessation of the I3 retractor neurons (Lu et al. 2013). Given that B43 activity comes after the major retractor motor neurons have stopped, it is possible that this activity also assists in protraction; i.e., helping return the odontophore to its rest position from its peak retraction. A complete description of the activity of the I1 motor pool requires further investigation beyond the scope of this work. Thus, in this model, we utilize B82 as the motor neuron for the dorsal and ventral I1 muscles and connect it such that it activates during protraction.

The extrinsic muscles E1, E2, and E6 were not present in either the previous *in silico* (Webster-Wood et al. 2020; Liao et al. 2021) or *in roboto* (Dai et al. 2022) biomechanics models, and thus required additional model neurons. Previous animal studies identified E1 and E6 as protractor muscles (Jahan Parwar and Fredman 1983), and thus, their motor neurons are active in protraction for all behaviors. E2 was identified as a retractor muscle (Jahan Parwar and Fredman 1983) and is here activated in retraction for all behaviors. The motor neurons for these extrinsic muscles have not previously been identified in the literature, but the motor neurons of other extrinsic muscles have been shown to reside in the cerebral ganglion rather than the buccal ganglia (Jahan Parwar and Fredman 1983). Following the naming convention of other cerebral ganglion neurons, here, the motor neurons for the extrinsic muscle Ex are labeled as C[Ex].

The additional B44/B48 motor pool and the phenomenological motor pools RU1, RU2, and RU3, which separate out the components of the B3/B6/B9 motor pool introduced in Dai et al. (2022) were not included here, as the muscles/muscle regions they innervate are not present in this model.

Finally, as in previous model iterations (Webster-Wood et al. 2020; Liao et al. 2021; Dai et al. 2022), interneurons and motor neurons receive proprioceptive feedback from the biomechanics. As signals from the biomechanics model are continuous (not discrete as in the Boolean network), these signals must first be thresholded. The “sensory neuron” outputs a 1 if the continuous signal is, for example, below a threshold, and 0 if it is above the threshold. These sensory signals can be activated by proprioceptive signals that are either greater than or less than a threshold, depending on the particular neuron. For a complete list of sensory thresholds, the directionality of the threshold, and the post-synaptic neuron receiving the signal, see Supporting Information Table 2. In this model, we include normalized grasper pressure and the relative position of the grasper in the head as proprioceptive signals. Normalized grasper pressure ($\tilde{P}_g = F_{I4}/T^{I4}_{max}$) provides feedback about the closure state of the grasper. To allow for more direct quantitative comparisons with animal data (Neustadter et al. 2007), including quantifying translation in terms of the distance from the jaw line to the front of the odontophore, in this model, the relative odontophore position was calculated as:

39 $$\begin{aligned} {\hat{x}} = \frac{(x_g + R) - (L_{head} + x_h) + 0.5(L^{I1v}_0 + L^{I1d}_0)}{0.5(L^{I1v}_0 + L^{I1d}_0)} \end{aligned}$$

where $L_{head}$ is the length of the rigid head, and $L_{head} + x_h$ is the current position of the jaw line. The factor $0.5(L^{I1v}_0 + L^{I1d}_0)$ is the average length of the I3 lumen at rest and was incorporated to normalize this term to be 0 when the odontophore is fully retracted and 1 when the odontophore reaches the jaw line. ${\hat{x}}$ is an affine transformation of the $x_{gh} = x_g - x_h$ parameter used in Webster-Wood et al. (2020) and is unitless.

### Computational methods and analysis

#### Model implementation

The model was implemented using Simulink (MATLAB r2024a, MathWorks). A variable step, variable order linear multistep numerical solver (*ode23*), was used to solve the dynamical system. Timesteps are restricted to be less than $10^{-1}$ s. For each timestep, the relative tolerance was set to $10^{-5}$, and the absolute tolerance was set to $10^{-4}$. To help minimize stiffness issues with friction models and abrupt changes of sign in muscle forces, zero-crossing detection was disabled. All simulations were run on an AMD Ryzen 5 5600X 6-core processor (3.70 GHz) with access to 16 GB of RAM. Under these conditions and using this hardware, we were able to solve the model in near-real time.

#### Model observables

The state variables that are calculated while solving the model are not always directly comparable with animal data. However, numerical correlates of the measurements taken during animal experiments can be calculated from these states. For scalar model observables that deal with individual cycles, values were calculated only for steady-state cycles. For time-continuous observables, values can be calculated for any cycle. To obtain these steady-state cycles (which, for stable regions of the parameter space, occurred after one start-up cycle), simulations were run for 20 seconds, as this was sufficient for all behaviors to cycle multiple times. The beginnings of cycles were identified by the transition of the B31/B32 neuron from *off* to *on*. The model observables that were compared to animal data are summarized in Table 1. For details about how these observables were calculated, see Supporting Information 1.

**Table 1:** Summary of scalar and continuous model observables. For calculation details, see Supporting Information 1

Name	Description	Calculation
*Scalar Observables*		
Cycle Time [s]	Duration of steady state behavior cycle	Time between sequential activation of B31/B32
Time in Protraction [s]	Duration of protractor motor neuron activity	Duration of B31/B32 activity
Percent Protraction [%]	Fraction of steady state cycle spent in protraction	Time in Protraction/Cycle Time
Translation Range of Motion [mm]	Distance traveled by the odontophore in one steady state behavioral cycle	$\text {max}(\Delta x) - \text {min}(\Delta x)$
Rotational Range of Motion [deg]	Angle traveled by the odontophore in one steady state behavioral cycle	$\text {max}(\theta _{obs}) - \text {min}(\theta _{obs})$
*Continuous Observables*		
Odontophore Translation [mm]	Time series of odontophore distance from jaws (more caudal is more negative)	$\Delta x \propto x_g + R - (L_{head}+x_h)$
Odontophore Angle [deg]	Time series of model radular stalk angle to the jaw line	$\theta _{obs} = \frac{180}{\pi }\left( \frac{\pi }{2} - (\theta _g - \theta _f )\right)$
Force on Seaweed [nominal]	Force applied to fixed seaweed by jaws or grasper	$F^{seaweed} = F^g + F^j$
Length of Ingested Seaweed [mm]	Amount of seaweed ingested during a behavioral cycle (negative for egestion)	$L_{ingested}(t) = \int _0^t \delta _{ingest}(\tau )\dot{\bar{x}}_f(\tau )d\tau$

#### Parameter estimation and tuning

In both the neural and biomechanical models, there are many parameters that must be estimated. The size of the parameter space means that many equivalent solutions may exist due to parameter coupling. This is especially true for model parameters that, if cast in arbitrary model units, could be scaled by any constant. To minimize the space of free parameters and the effect of arbitrary parameter scaling, we estimated as many parameters as possible from independent measurements in the existing literature. For the complete details of the model tuning, see Supporting Information 2, but we briefly summarize the process here.

The anatomical parameters were calculated using the fixed buccal mass image presented in Neustadter et al. (2007). This was also used to set the units of length in the model by constraining one model length unit to be equal to the measured radius of the odontophore. Muscle parameters for the I2, I1/I3, I4, and hinge were based on models and data from Yu et al. (1999), Sukhnandan et al. (2024), Morton and Chiel (1993), and Sutton et al. (2004a), respectively. All timing parameters were normalized to the activation time constant of I2 based on the results of Yu et al. (1999), and the magnitude of this value (in absolute units, i.e., seconds) was set to achieve appropriate behavioral durations. These behavioral durations were used to set the absolute units of time, thus disallowing any arbitrary scaling of the time constants and corresponding neural activity durations. The remaining muscle parameters and the feedback thresholds in the neural model were hand tuned, first to produce multifunctional behaviors and then to match internal kinematic data. Only the magnitudes of muscle forces were left in arbitrary model units. However, because scaling all parameters containing units of force by the same constant would not change the dynamics, and because we perform no quantitative comparison of the force to animal data, this lack of absolute force units is not consequential to the model. However, to minimize the size of the parameter space, we did fit the relative magnitude of forces to animal data where possible (See Supporting Information Section 2). All parameters used in this model can be found in the Supporting Information Section 4 and the provided code repository.

#### Animal data

Animal datasets were extracted and digitized from various literature sources to enable quantitative comparisons and assessment of model performance across all behaviors of interest. All time series data were digitized by hand using WebPlotDigitizer v4 (Rohatgi 2022). Internal kinematics data were collected from Neustadter et al. (2007) for biting and swallowing and from Novakovic et al. (2006) for rejection. Neustadter et al. (2007) reports two different angles for the odontophore, one from the jaw line to the posterior edge of the I6 muscle, and one from the I6 to the radular stalk axis. Because the model presented here does not have geometry corresponding to the I6, we cannot compare directly to these data. Therefore, we calculated the angle from the jaw line to the radular stalk axis from these animal data to compare to the model observables. These data correspond to cycles from 2 animals (total of 4 cycles). Novakovic et al. (2006) reports a rotation for rejection, but which angle is reported is unclear. Therefore, only odontophore translation data were extracted from this paper. These data were from 1 cycle from 1 animal. To allow the kinematics data to be aligned and resampled for comparison with the model data, the animal data were fit to an $8^\text {th}$ order Fourier series. The Fourier series was fit to three concatenated duplicates of the single animal cycle to minimize the Gibbs phenomenon at the beginning and end of the cycles. This was performed for both the mean and standard deviation (if present) curves. These kinematic data from Neustadter et al. (2007) and Novakovic et al. (2006) were also used to calculate the translational and rotational range of motion (ROM) for the animal.

Neuromechanical data were extracted for swallows on untethered and tethered seaweed strips (referred to as unloaded and loaded swallows) from Gill and Chiel (2020), and data for the length of ingested seaweed during swallowing were extracted from Lum et al. (2005). As with the kinematic data, we interpolate these data to allow for resam-pling at time points corresponding to our model data. The neuromechanical data were generally not continuously periodic, with prolonged periods where the neural signals were zero, and the length of ingested seaweed data were strictly aperiodic. Therefore, a Fourier interpolation would not be appropriate. Instead, these data were spline interpolated to allow for resampling. Specifically, we used a cubic spline interpolation implemented using the MATLAB function *interp1* with the ‘method’ specified as ‘spline’ (MathWorks, r2024a).

Timing and duration of protraction data for the different behaviors were collected from across multiple papers. Biting data was taken from experiments that led to Cullins et al. (2015) but were not published in that study ($N=7$ animals). Data for unloaded swallowing were taken from both Cullins et al. (2015) and Gill and Chiel (2020) ($N=12$ animals total), and data for loaded swallowing were taken from Gill and Chiel (2020) ($N=5$ animals). For these datasets, mean and standard deviation data were available for multiple individual animals. To aggregate these data into single estimates of an “average *Aplysia*” in a way that incorporates the different number of samples per animal in the assembled datasets, a bootstrap resampling was conducted to obtain sampling distributions for the population-level mean and standard deviation (See Supporting Information 3.1). Data for rejection was digitized from plots of neural signals in Ye et al. (2006) that aggregated data from 5 animals. Data were collected specifically for Type B rejections, as these are characterized by large egestion of seaweed similar to what is seen in the model. The start of the cycle was estimated as the beginning of I2 activity, and the end of the cycle was estimated to be the cessation of activity of the buccal nerve 2 (BN2) third largest unit (Ye et al. 2006), which likely reflects the activity of B43 (Lu et al. 2013). The cycle length for rejection was estimated as the time between the start of I2 activity and the end of BN2 activity, with the standard deviation being calculated from the standard deviation in these values. The time at which I2 activity ended was also recorded to calculate the duration of protraction.

#### Statistical analysis

To validate this model with animal data and to investigate where additional model improvements may be needed, we conducted a quantitative comparison between the model observables and previously reported animal data. For time-varying signals, we calculated both the cross-correlation at zero lag and the root mean square error (RMSE). These signals include the translation and rotation kinematics for biting, unloaded swallowing, and rejection. We also calculated the cross-correlation between model and animal data for the swallowing neuromechanics reported by Gill and Chiel (2020). However, because the neural signals in the model and animal data report different quantities (neuron state for the model and median firing frequency for the animal data), the RMSE would not be meaningful. The cross-correlation was calculated at zero lag because signals were already aligned by either kinematically- or neuromechanically-relevant features in the signal. For the scalar observables, we were able to conduct statistical tests to compare model outcomes with animal data. When validating neuromechanical models, it is important for the predictions to be both statistically equivalent to and not statistically different from the animal data (Robinson and Froese 2004). Both assessments are needed in this context because results being not statistically different does not inherently imply statistical equivalence (Lakens 2017). Therefore, we conducted both t-tests to test for mean differences and equivalence tests, which we will now describe. One-sample tests were conducted because the model is fully deterministic and, therefore, has no distribution associated with its observables. For both test types, significance was set at $\alpha =0.05$. To take advantage of the bootstrapped distributions (See “Animal data”), we conducted the statistical tests based on the confidence interval on the metric of interest. For the t-test, the 95% confidence interval was calculated, and if the interval does not contain 0, then there exists evidence for a difference between the means at the 5% level (Lakens 2017). For the equivalence test, the 90% confidence interval was calculated, and if the confidence was fully contained within a prespecified equivalence bound ($[-d,d]$) expressed in the form of a Cohen’s *d* effect size, then this was taken as evidence that the means were equivalent up to a difference of $\pm d$ (Lakens 2017). We determined an appropriate equivalence bound by conducting a power analysis and choosing the bound that we could detect at a power of $1-\beta = 0.8$. For details of the power analysis, see Supporting Information 3.2. This analysis was conducted for each of the different behaviors because different sample sizes were available. For both loaded swallowing and rejection, there were data for $N=5$ animals, so power was achieved at an equivalence bound of $d=1.65$. For biting ($N=7$), power was achieved at $d=1.27$, and for unloaded swallowing ($N=12$), power was achieved at $d=0.92$. These were the bounds that were utilized for the equivalence tests in this work. These tests were conducted for Cycle Time and Time in Protraction, but not for Translational and Rotational Range of Motion, as these metrics only had data for either $N=1$ or 2 animals.

#### Code and data availability

All code and data are available for download on GitHub (Link to Model Code), and an archived version is available through Zenodo (doi: 10.5281/zenodo.15699753). All code is provided in MATLAB R2024a.

## Results

### Model qualitatively produces multifunctional feeding behavior

**Fig. 5:**
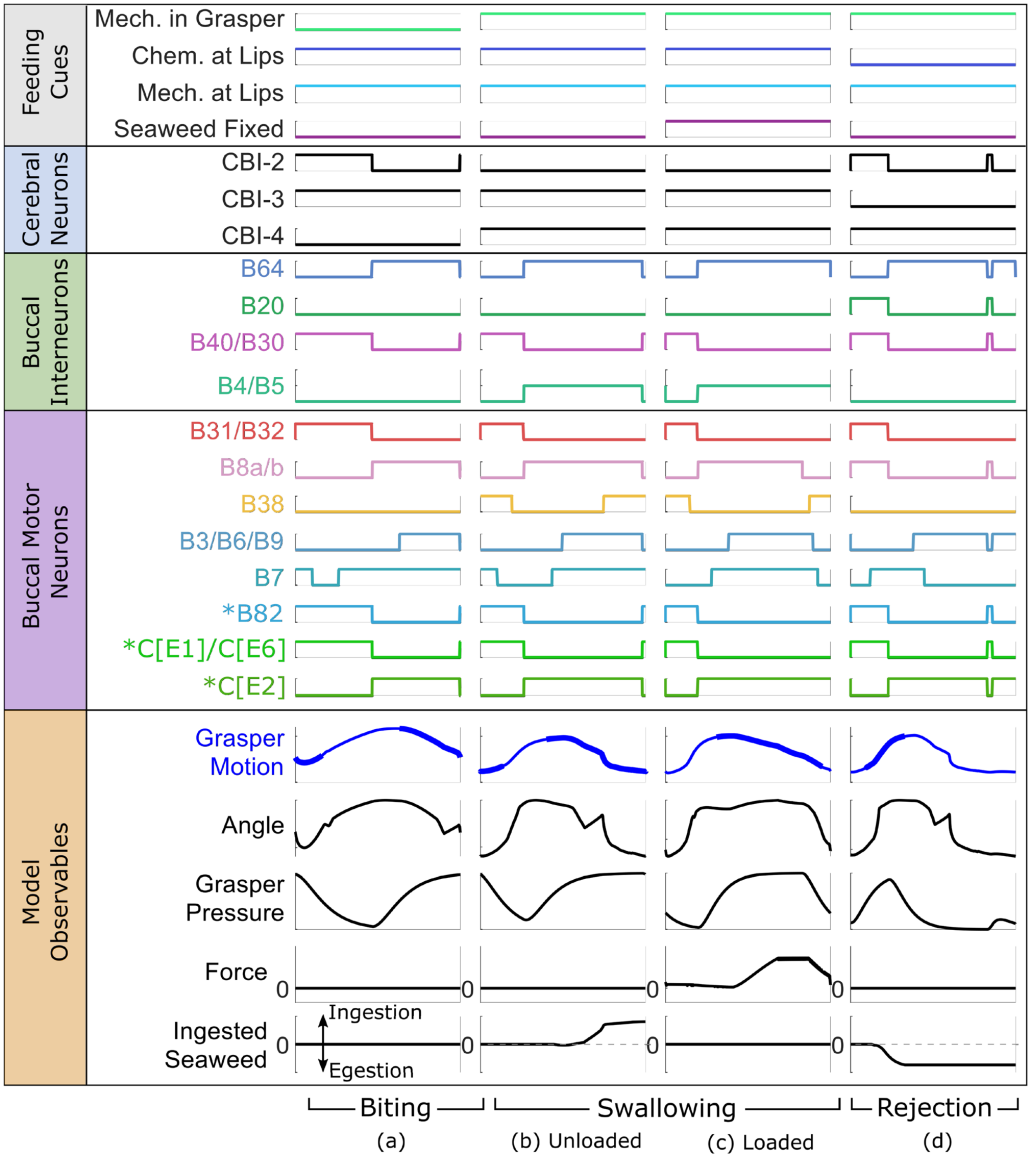
Steady state simulation results for (**a**) biting, (**b**) unloaded swallowing (untethered seaweed), (**c**) loaded swallowing (tethered seaweed pulled taut), and (**d**) rejection. Steady state was achieved after one start up cycle for all behaviors. Signals are grouped according to Fig. 4. The signals for all model neurons indicate their current Boolean state (0 or 1). These states abstract the true dynamical nature of these neurons’ responses during behavior (see Webster-Wood et al. (2020) Figure 2 for a useful side-by-side comparison of neural recordings and Boolean representations). Each behavior is generated by providing different feeding cues to the model. The bolded line in the Grasper Motion signal indicates when the grasper is closed ($F^{I4}>0.5F^{I4}_{max}$). Dashed line in the Ingested Seaweed signal shows the level of no ingestion. Asterisks (*) indicate neurons that were not in the previous Webster-Wood et al. (2020) model and were added here

Compared to previous neuromechanical models of *Aplysia* feeding, the biomechanical model presented here increases the degree of anatomical and mechanical agreement with the animal system. Compared to previous models, several previously abstracted structures have been explicitly modeled, and behaviorally relevant degrees of freedom, specifically the rotation of the odontophore and the length of the I3 lumen, have been incorporated. With this additional complexity, the model successfully reproduces the multifunctional behaviors demonstrated by *Aplysia* and by previous models when presented with different mechanical and chemical stimuli (Fig. 5). This model can approximately capture both the neural activity of the control ganglia, represented as Boolean signals (for a comparison between recorded neural activity and the corresponding Boolean framework representation, see Webster-Wood et al. (2020) Figure 2), and various kinematic and behavioral observables. Biting responses were elicited when the model was presented with mechanical and chemical stimuli at the lips but not at the grasper. When the grasper was also presented with mechanical stimulus, the model instead performed a swallowing behavior. Finally, when the chemical stimulus was removed from the lips but a mechanical stimulus persisted in the grasper, instead of ingesting food, the model egested food in a rejection-like behavior. These behaviors were achieved by changing the chemical and mechanical sensory cues while only requiring a single, constant set of tuned parameters for the combined neuromuscular system.

**Fig. 6:**
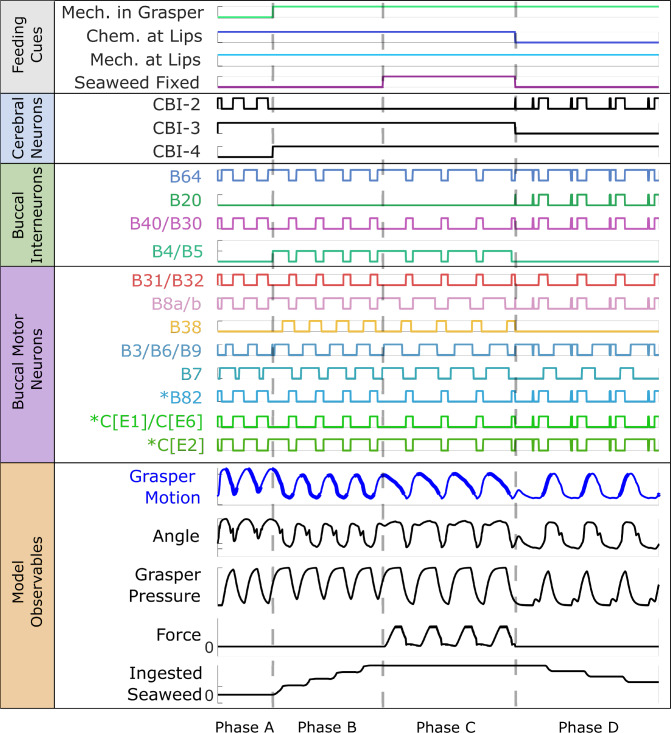
Behavioral switching. If the feeding cues are changed mid-simulation, the model can respond and successfully switch to a different steady-state behavior. For this simulation, the model is presented with food (Phase A), resulting in a biting behavior. Mechanical stimulus is then provided to the grasper (Phase B), causing a shift to an unloaded swallowing behavior. Then, the food becomes fixed (Phase C), and the model shows a loaded swallowing behavior. Finally, the chemical stimulus is removed from the lips of the model but mechanical stimuli continued to presented to the grasper (Phase D), and the model switches to a rejection behavior. Dashed gray lines show the dividing lines between the phases when the feeding cues are changed, but all results are from a single continuous simulation. Data is presented in the same manner as in Fig. 5 (see its caption for more details)

The model also successfully demonstrated the ability to adapt to changing environmental cues and maintain robust control of the feeding behaviors. This is seen in the model’s ability to change behaviors when sensory cues are switched mid-simulation (Fig. 6). During this simulation, the model is first presented with food but not allowed to grasp it (Phase A), during which time it performs a biting behavior. Then the model was allowed to begin pulling in the food (Phase B), at which point it switched to a swallowing behavior. As it continued to pull in food, the food was suddenly fixed in place (Phase C), and the model continued attempting to swallow, now generating force on the fixed food. Finally, the chemical cue indicating that the object on which it is feeding is edible was removed while a mechanical stimulus continued to be presented to the grasper (Phase D), at which point the model began rejecting the food and pushing it back out of the buccal mass.

**Fig. 7:**
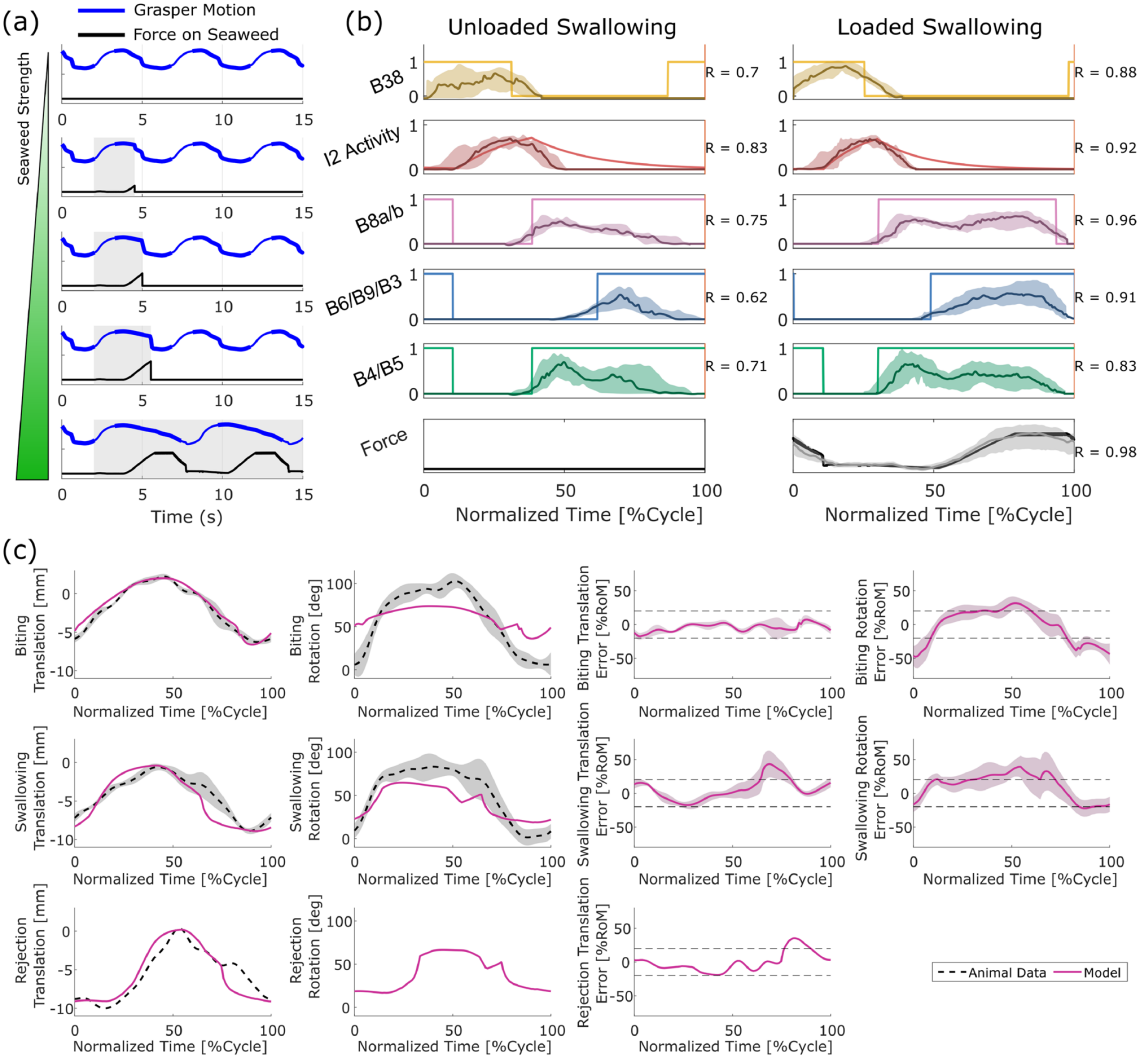
Computational experiments and comparison to animal data. (**a**) Feeding on finite strength seaweed. Each plot presents the grasper motion (blue) and force on seaweed (black) results for simulations with increasing strength of seaweed. Bolding of the grasper motion line indicates that the odontophore is closed, and the shaded gray region indicates when the seaweed is fixed (and not broken). As the force on seaweed reaches the failure strength of the seaweed, the food becomes unfixed and the model returns to an unloaded swallowing behavior. (**b**) Neuromechanics of unloaded and loaded swallowing behavior compared to animal data from Gill and Chiel (2020). Jagged lines and shaded regions show the median and interquartile range, respectively, of the animal data. The binary signals for B38, B8a/b, B6/B9/B3, and B4/B5 show the model neural activity while the smooth lines for I2 activity and Force show the activation level of I2 ($A_{I2}$, see Equation 20) and the force applied to seaweed, respectively, from the model data. The cross-correlation (R) was calculated for each signal. Signals were aligned to the beginning of I2 activity. (**c**) Comparison of model observables and internal kinematics as reported in Neustadter et al. (2007) for biting and swallowing and in Novakovic et al. (2006) for rejection. Fuchsia lines show the model data, and black dashed lines and shaded regions show the mean ± 1 standard deviation animal data. Columns 1 and 2 show the odontophore translation and rotation, respectively, for the model and animal. Columns 3 and 4 show the error between the model and animal kinematics, normalized to the range of motion (ROM), for translation and rotation, respectively. The gray dashed lines indicate an error of ±20% of the ROM. For rejection behaviors, only one translation cycle was available, so the standard deviation was not reported, and no rotation data was reported

Behavioral robustness can also be seen in simulated feeding experiments where the food suddenly breaks and is no longer fixed (Fig. 7a). During these tests, the model begins feeding on unfixed seaweed. Then, at t = 2 s in the plots, the seaweed becomes fixed in place. However, unlike in previous simulations with fixed seaweed, here the seaweed has finite strength. If the force on the food exceeds that strength threshold, the food breaks and becomes unfixed again. Seaweed strengths were incrementally increased from breaking at 0 force to not breaking within the range of forces that the model generates on unbreakable seaweed. Throughout this range of strengths, the model could recover from the sudden change in boundary condition by the end of the current cycle and successfully switch back to an unloaded swallowing behavior. As with previous models (Webster-Wood et al. 2020), this is an emergent behavior of the network and not a programmed behavior. Additionally, as seen both in behaving animals (Gill and Chiel 2020) and in the previous model (Webster-Wood et al. 2020), with increasing strength of the seaweed comes an increase in the cycle length until it can no longer break the seaweed. However, the kinematics predicted by this model do differ during this break from the previous model. In the model presented in Webster-Wood et al. (2020), the kinematics remain smooth through the transition from fixed food to unfixed food, with no sudden change in odontophore position. Here, the sudden change in force is accompanied by an initial sudden backward change in the odontophore position before returning to a smooth cycle. The magnitude of this change also increases with increasing seaweed strength. This difference is predominantly due to the increased damping in the previous model. Previously, the level of damping, characterized by the time constant of the dynamics, in normalized model units was set to 1. Here, the level of damping is much lower, set at a value of 1/50 in normalized model units. Thus we would expect the previous model to exhibit a far more damped response. Without access to internal kinematics data measured during similar types of experiments, we cannot ascertain whether this transition in the animal is rapid or smooth. If the transition in real animals is smooth, it may point to other mechanisms besides passive damping that act to decelerate the odontophore.

The model generally produces qualitatively similar steady-state results to those observed in animal experiments, providing support for the assumption that these behaviors can be captured solely through the midsagittal mechanics (A1). However, there are two key areas where, even in preliminary comparison, we saw mismatches with the animal behavior, which contradict our assumption regarding the geometry of the buccal mass muscles (A2). First, during rejection behavior in the animal, it is seen that the multi-action neurons B4/B5 fire strongly at the onset of retraction (Ye et al. 2006). This firing is stronger than their activity in other behaviors, which motivated the use of a ternary variable for its firing state in this and previous neural models (Webster-Wood et al. 2020; Dai et al. 2022), instead of a standard binary state that is characteristic of a standard Boolean model. It has been demonstrated experimentally that this strong firing prevents the premature closing of the jaws and I3 muscle complex, which would prevent the successful retraction of the grasper in its open state (Ye et al. 2006; Webster-Wood et al. 2020). This retraction, therefore, would have to be mediated by a different muscle group, namely the hinge. However, B4/B5 does not fire during rejection behavior in the model using the current parameter tuning (Fig. 5d), and retraction is primarily due to forces from the I3 muscle. If the tuning is changed so that B4/B5 fires during rejection, delaying the firing of B3/B6/B9, a much worse agreement was seen in the kinematics (Fig. 7c). The second mismatch also deals with an over-reliance on the I3 muscle group. During biting behaviors in animals, the motor neurons B6/B9 do fire during the retraction phase, but the frequency of firing is insufficient to generate significant forces in I3 (Lu et al. 2015). Instead, retraction in biting may also be predominantly mediated again by the hinge (Sutton et al. 2004a). In contrast, the model relies heavily on the I3 for generating retraction forces in biting, as seen by the long period of firing of B3/B6/B9 (Fig. 5a). If this firing is turned off in the model, the system does not cycle, and the odontophore becomes stuck in its forward position. These both point to shortcomings in the modeling of the hinge structure, which does not appear to be adequately approximated by simple line element geometries.

**Table 2:** Quantitative comparisons between model steady-state cycles and averaged animal kinematics (Neustadter et al. 2007; Novakovic et al. 2006)

Behavior	Cross-Correlation		Root Mean Squared Error	
	Translation	Rotation	Translation [mm]	Rotation [deg]
Biting	0.985	0.937	0.609	24.1
Unloaded Swallowing	0.977	0.978	1.39	18.3
Rejection	0.974	–	1.48	–

	Translational Range of Motion [mm]		Rotational Range of Motion [deg]	
	Model	Animal Mean [STD]	Model	Animal Mean [STD]
Biting	8.61	8.50 [0.45]	37.7	96 [15]
Unloaded Swallowing	8.52	8.29 [0.57]	46.2	81 [17]
Rejection	9.30	10.4 []	–	–

### Model comparison with animal data

The model was quantitatively and statistically compared to previously reported animal data using both time-varying and scalar metrics. The model agrees well with the internal kinematics reported in Neustadter et al. (2007) (Table 2, Fig. 7c). For both translation and rotation in all behaviors, the cross-correlation was greater than 0.9, and the RMSE for translation was less than 1.5 mm. Additionally, for biting, the model was within ± 20% of the range of motion (ROM) throughout the full feeding cycle (Fig. 7(c, Column 3)). This was also true for swallowing and rejection, except for small portions of the cycle. In unloaded swallowing, 14% of the cycle had an error greater than 20%, with a maximum error of 43%. For rejection, 12% of the cycle exceeded 20% error, with a max error of 35%. However, the model dramatically underpredicts the rotational range of motion, particularly in biting, where the model achieved less than half of the rotational ROM of the animal. The agreement is better in swallowing but was still 2 standard deviations lower than observed in the animal. We believe this discrepancy is due to limitations in both the mechanics and anatomy of the hinge and the odontophore (See Sections 4.1.3 and 4.1.4). This comparison could not be conducted in rejection with the available animal data.

For unloaded and loaded swallowing, the neuromechanics of the model were compared with data from Gill and Chiel (2020), and the cross-correlation was calculated (Fig. 7b). In general, the cross-correlations were high, with all but one of the signals scoring $R\ge 0.7$. These high cross-correlation values indicate an appropriate capturing of the phasing of neural activity in the animal. However, the use of Boolean neurons in this model limits our ability to capture the finer dynamical details present in the animal data (See Section 4.1.1). The largest disagreements exist in the B6/B9/B3 signal in unloaded swallowing ($R=0.62$). This discrepancy relates to the model being under-constrained in protraction. To achieve the correct kinematics, the parameters had to be tuned such that the B31/B32 motor neurons, which innervate I2, turned off partway through protraction. This allowed the odontophore to slow down before reaching peak protraction, at which point the B6/B9/B3 motor neurons activated, generating force in I3 for retraction. Otherwise, the slope of the translation would be too high. However, for this to occur and for the odontophore to still achieve the appropriate range of motion, the firing of the I3 motor neurons needed to be delayed. If there were other mechanisms to slow down protraction, this delay would not need to occur.

**Fig. 8:**
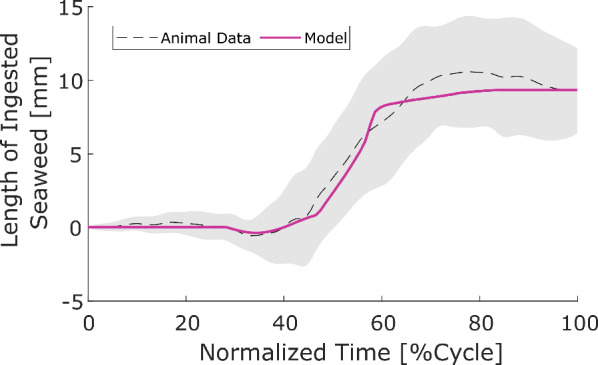
Length of seaweed ingested in a single swallowing behavior. The gray dashed line and shaded region show the animal data extracted from Lum et al. (2005), and the fuchsia line shows the model data

The model was also compared to external data for swallowing behavior in the form of the length of seaweed that was ingested in a single swallow (Lum et al. 2005) (Fig. 8). Here, the model performed very well, with a cross-correlation of $R=0.996$ and an RMSE of 0.70 mm. The model was not explicitly tuned to match this curve, but rather, this agreement arose because of the timing and kinematic agreement with animal data. As in the animal, the position of the seaweed does not change during the beginning of protraction, as it is held in place by the jaws (i.e., the pinched anterior of the I3 muscle). Near the peak of protraction, the jaws open, and food is slightly egested before the odontophore changes direction. At this point in the animal data, the food is pulled in towards the esophagus at a roughly constant rate before coming to rest at peak retraction. The rate of seaweed ingestion in the model is not as uniform as in the animal, with a period of more rapid ingestion followed by a period of very slow ingestion before coming to rest. Despite these differences, the model approximates the timing of seaweed ingestion and the length of seaweed ingested within a single swallow well.

**Table 3:** Scalar metric comparison with animal data. Animal data were aggregated from previous literature (Cullins et al. 2015; Gill and Chiel 2020; Ye et al. 2006)

Behavior	Animal Value (Mean ± SEM) [STD]	Number of Animals	Model Value	%Difference [Effect Size]	Equivalent?	Different?
Cycle Time [s]						
Biting	4.36±0.30 [0.77]	7	4.38	−0.36 [−0.02]	Y	N
Unloaded Swallowing	4.84±0.29 [0.98]	12	4.91	−1.29 [−0.06]	Y	N
Loaded Swallowing	–	–	6.37	–	–	–
Rejection	8.05±0.38 [0.85]	5	6.99	13.2 [1.24]	N	Y
Percent Protraction [%]						
Biting	48.3±2.4 [5.9]	7	46.4	3.92 [0.32]	Y	N
Unloaded Swallowing	28.7±2.4 [7.6]	12	26.2	8.63 [0.33]	Y	N
Loaded Swallowing	18.5±1.8 [3.8]	5	19.0	−2.29 [−0.11]	Y	N
Rejection	37.3±3.4 [7.6]	5	23.6	36.8 [1.80]	N	Y
%Increase in Cycle Length (relative to unloaded swallowing)						
Loaded Swallowing	32.9±10.1 [20.9]	5	29.9	9.19 [0.14]	Y	N

**Fig. 9:**
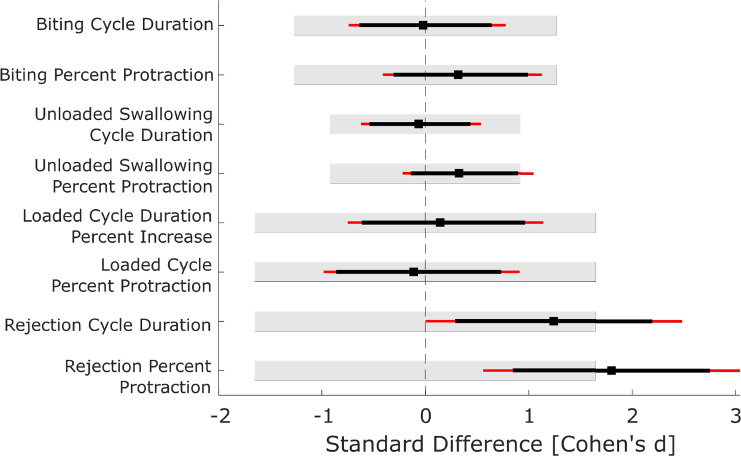
Statistical comparison between model predictions and animal data. Square marks show the normalized mean difference; black lines show the 90% Confidence Interval; and red lines show the 95% confidence interval. The gray-shaded region indicates the equivalence bound for the given metric. Positive standard differences indicate that the mean animal value is greater than the model value. Equivalence is supported if the 90% confidence interval is fully contained within the equivalence bound, which was true for all but the two rejection metrics. If the 95% confidence interval intersects the 0 difference line (dashed gray line), then the t-test fails to reject the null hypothesis

Positive percent differences indicate that the animal mean is greater than the model value. The Equivalence column presents the results of the equivalence test (Y: 90% confidence interval is fully within the equivalence bound, see Fig. 9), and Difference column presents the results of the t-test (Y: 95% confidence interval does not intersect a standard difference of 0)

Finally, for all behaviors of interest, a statistical comparison was conducted for the length of behaviors and the fraction of behavior that was spent in protraction (Table 3, Fig. 9). Specifically, both equivalence tests and t-tests were conducted to determine if the model predictions agree with the animal data. For loaded swallowing, the raw cycle time was not compared to animal data, as the animals in this dataset skewed towards longer unloaded swallows and thus may not be representative of the whole population (See Supporting Information 3.3). However, the length of loaded swallows relative to unloaded swallows for a given animal may be a more consistent metric, so this was compared to model predictions. For all metrics related to biting, unloaded swallowing, and loaded swallowing, the model was statistically equivalent to and not statistically different from the animal data (Fig. 9, Table 3), suggesting that this model adequately represents an “average *Aplysia*” in these behaviors. However, both metrics for rejection were non-equivalent, with both the cycle duration and percent protraction being significantly lower than the mean animal value. This disagreement relates to the underperformance of the hinge and the under-constraining of the odontophore in protraction. As with swallowing behaviors, the rate of protraction was too fast compared to animal data. To achieve the correct time-normalized kinematics, the rate of retraction had to be similarly increased. This was achieved by activating the I3 muscle at the peak of protraction rather than waiting for the hinge to initiate retraction, as is seen in the animal (Ye et al. 2006). If B4/B5 were to fire, delaying the firing of the B3/B6/B9 motor pool, the odontophore would sit near peak protraction for a prolonged period, only moving slightly because of how little force the hinge could generate. This would allow the cycle duration of rejection to better approximate the measured value but at the cost of a much worse kinematic match. The under-constraining of protraction in this model provides evidence against our assumption that the bulk tissue passive forces may be neglected (A3).

### Comparison with previous biomechanical model

**Fig. 10:**
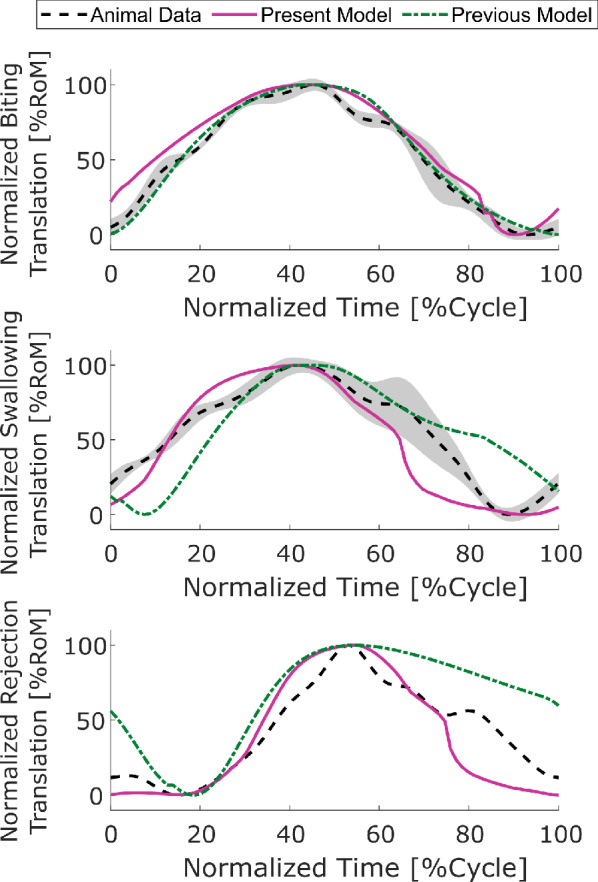
Comparison with previous biomechanical model. Animal data (biting and swallowing (Neustadter et al. 2007), rejection (Novakovic et al. 2006)) is presented as black dashed lines (standard deviation shown by the shaded region). Data from the new model is shown in the solid fuchsia line, and the previous model (Webster-Wood et al. 2020) is shown in the dot-dashed green line. Because the mechanics of the previous model only had nominal units, this comparison could not be conducted in real-world units. Instead, kinematic trajectories were normalized by the range of motion for each behavior. Both models capture the kinematics of biting very well (top), while the new model performs better in swallowing (middle) and particularly in rejection (bottom). Cross-correlation and RMSE values are reported in Table 4

We performed a quantitative comparison with the previous state-of-the-art biomechanical model (Webster-Wood et al. 2020) to investigate if the additional computational complexity, and biological realism, of our new model provided substantial improvements (Fig. 10). To do this, we calculated both the cross-correlation and RMSE for the present and previous models relative to the animal’s internal kinematics. However, because the previous model had no geometry, this comparison could not be conducted in real-world units. Instead, the odontophore translation was normalized to the range of motion of each behavior, and these normalized curves were used to compare the models. The previous model already captured biting behavior very well, with a cross-correlation of $R=0.997$ and RMSE of 5.62%, and the new model performed similarly well ($R=0.993$, RMSE = 9.63%). However, the new model improved the kinematics of both unloaded swallowing and rejection, with the RMSE decreasing from 21.89% to 16.3% and from 25.78% to 17.0%, respectively, compared to the previous model. The new model also better predicts the ratio between biting and swallowing cycle durations, with a 0.9% error in the new model compared to a 14.7% error in the previous model. Finally, the new model underpredicts the rejection to biting timing ratio, with an error of −13.5% compared to the previous model’s 11.2% overestimation.

**Table 4:** Quantitative comparison to the previous biomechanical model presented in Webster-Wood et al. (2020)

Behavior	Normalized Translation Cross-Correlation		Normalized Translation RMSE		Behavior Duration Ratio (Relative to Biting)		
	Current Model	Previous Model	Current Model [%]	Previous Model [%]	Animal DataMean±STD	Current Model [%Error, d]	Previous Model [%Error, d]
Biting	0.993	0.997	9.63	5.62	–	–	–
Unloaded Swallowing	0.967	0.942	16.3	21.89	1.11±0.30	1.12 [0.9%, 0.03 STD]	1.27 [14.7%, 0.55 STD]
Rejection	0.949	0.963	17.0	25.78	1.85±0.38	1.60 [−13.5%, 0.66 STD]	2.05 [11.2%, 0.54 STD]

### Time constants converge to *in vivo* measurements with no damping

**Fig. 11:**
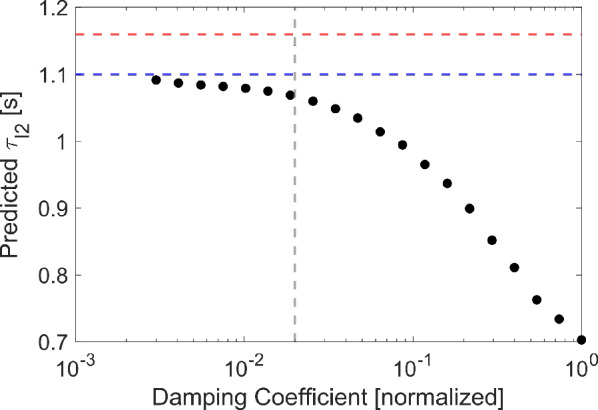
Convergence of time scaling with decreasing damping parameter. As the magnitude of the damping parameter, plotted in normalized model units, decreases towards 0 (no damping), the predicted value for the I2 time constant converges to a value of $\sim$1.1 s. Black circles show the results of individual simulations. The red dashed line shows the measured value of the I2 activation time constant of 1.16 s (Yu et al. 1999) (See Supporting Information 2.1). The blue line shows the converged value of the predicted I2 time constant. Finally, the vertical gray line shows the level of damping that was chosen for the final tuning

During the tuning of the model, all parameters with units of time were normalized to the I2 time constant (i.e., in model units, $\tau _{I2}=1$). Then the value of the I2 time constant (in absolute units) was scaled such that the behavior durations of the model behaviors were in line with the times observed in animals. As all time-dependent values were uniformly scaled by this value, this acts only as a change of units and does not affect the dynamics of the system. Thus, based on animal behavior duration, we were able to make an independent prediction of the I2 time constant, in contrast to previous biomechanical models (Webster-Wood et al. 2020; Liao et al. 2021) where the damping parameter was set at 1 and not tuned. However, during the tuning of this model, we discovered that to obtain the correct magnitude of translation in the correct amount of time (relative to animal data), the damping parameter had to be significantly lowered. For the main results presented here, that damping parameter was empirically set at 1/50 in model units. To examine how the model would behave in the no-damping limit, we conducted a sweep of damping parameters logarithmically spaced from 1.26 down to 0.0017 (Fig. 11). Below this value, the model experienced numerical instabilities due to the stiffness of the system and the specified tolerances of the solver. For each damping coefficient, biting behavior was simulated with an I2 time constant equal to 1, and the cycle time was calculated. Then, the required time scaling (and therefore the predicted value of $\tau _{I2}$) was calculated as the mean animal bite time divided by the measured cycle time. As the value of the damping coefficient approached 0, the predicted value of $\tau _{I2}$ approached a value of 1.1 s. From our fitting of the double-first-order filter model to the I2 activation function found in Yu et al. (1999), we calculated a double-first-order filter time constant of 1.16 s for the animal data. This is in very good agreement with the value converged to by the model. This supports the idea that this model could be used to predict biological parameters by fitting to gross kinematic measures. Moreover, it provides additional evidence that damping does not play a significant role in shaping behaviors in this system. Because of this, damping may be removed in future iterations of this model, reducing the “dynamics” to a $0^{\text {th}}$ order system, as has been proposed previously (Kundu et al. 2022).

## Discussion

The model presented here was largely successful in both qualitatively and quantitatively matching animal behavior. The three primary feeding behaviors generated by the animal– biting, swallowing, and rejection– could be reproduced by switching the sensory cues provided to the model (Fig. 5), and the kinematics of these behaviors generally agreed well with internal kinematics measured in the animal (Fig. 7c). The model can also automatically switch between behaviors when these sensory cues are changed (Fig. 6). Compared to previous biomechanical models that were tuned solely to achieve multifunctionality, here, the model achieved both multifunctionality and quantitative agreement with animal data. This quantitative agreement to kinematics data was similar to or better than previous biomechanical models when comparing normalized data (Fig. 10, (Webster-Wood et al. 2020)). Because this new model incorporated explicit geometry, much closer to the real kinematics of the buccal mass, comparisons to animal data could also be made in real-world units (Figs. 7c and 8, Table 3). The scalar metrics observed from the model were statistically equivalent to the measured animal data for both biting and swallowing behaviors (Fig. 9), though the model underpredicted the timescale of rejection behaviors.

Through tuning this model to kinematics and timing data, an independent estimate of the I2 time constant could be made, with this estimate agreeing with animal data within 5.5% (Fig. 11, (Yu et al. 1999)). The agreement with animal data was achieved using both parameter estimation to existing animal datasets and *ad hoc* hand-tuning. Even better performance could be achieved using numerical optimization techniques (Wang et al. 2022b; Santos and Valero-Cuevas 2004).

### Limitations

#### Choice of neural and muscle model

In our model, the neurons are modeled as binary switches instead of as continuously varying dynamical systems. This simplified model was successful in producing steady-state behaviors as demonstrated here and previously (Webster-Wood et al. 2020; Liao et al. 2021; Dai et al. 2022), but it is limited in its ability to investigate the effects of non-bursting neural signals in control (Lu et al. 2015), intermediate behaviors not coded in the cerebral neuron layer, such as rasping (Gill and Chiel 2020), and dynamical processes like neuromodulation (Evans et al. 1996; Hurwitz et al. 2000; Cropper et al. 2019). It also cannot replicate the variability in the neural signals, which leads to cycle-to-cycle variability observed in animals (Kupfermann 1974; Cullins et al. 2015; Lum et al. 2005). Finally, the Boolean model’s ability to only capture “fully on” or “fully off” neural activity may be contributing to some of the mechanical disagreement in the model. In multiple behaviors, the model-predicted protraction is occurring too quickly. While this is likely predominantly related to the failings of the hinge and the lack of a passive I3 force (see “Muscles are more than active line elements”), this could also be related to an overestimation of the I2 force. The I2 motor neurons fire at between 12-17 Hz during *in vivo* swallowing behaviors (Gill and Chiel 2020), but the maximal force in I2 is not achieved until closer to 25 Hz stimulation (Yu et al. 1999). Therefore, we would not expect the I2 to generate its maximal force during swallowing behaviors. Because the maximal force of I2 in the model was set using the maximal force measured in Yu et al. (1999), a Boolean input of 1 would cause the I2 muscle to approach its maximal force, rather than an intermediate force associated with a stimulation frequency of $\sim$15 Hz. Utilizing a neural model with spiking neurons or a continuous rate-coded output may help alleviate these issues.

In addition to the simplified neuron models, the muscle models implemented here, with their linear passive response and length-independent active force components, greatly abstract the properties of biological muscle. In reality, both vertebrate and invertebrate muscles are highly nonlinear materials in their passive response, their active properties, and their dynamics. The active force generated by muscle is dependent both on its length and its shortening velocity (Zajac 1989). Molluscan muscle, acting more akin to vertebrate smooth muscle than skeletal muscle, can demonstrate a catch phenomenon, in which muscle maintains tension without additional neural activation (Butler and Siegman 2010). At a molecular level, the tension in muscle is initiated by nonlinear dynamical calcium signaling pathways and developed by nonlinear reaction kinetics in the cross-bridge. Many of these properties are dependent on neuromodulatory effects (Brezina et al. 2003; Hurwitz et al. 2000; Evans et al. 1996).

Different combinations of these nonlinearities have been captured in various models of invertebrate muscle (Brezina et al. 2003; Yu et al. 1999; Sukhnandan et al. 2024) and invertebrate neuromechanical systems (Sutton et al. 2004b; Wang et al. 2023), and these nonlinearities can have meaningful impacts on the behavior of these tissues and the organisms they comprise. By capturing nonlinearities in the calcium dynamics, Wang et al. (2023) were able to provide a mechanistic explanation for the dual time constants of muscle contraction in *Hydra*. Harischandra et al. (2019), through a systematic comparison of various linear and nonlinear models of the neuromuscular transform and force production, demonstrate that, in insect muscle, nonlinearity was necessary to account for the differences in twitch and tetanic responses, and that these nonlinearities are particularly important in behaviors where muscle stimulation frequency is low. Because the *Aplysia* feeding system can be modeled as quasistatic (Sutton et al. 2004b; Rogers et al. 2024), and thus forces related to inertia and viscosity can be considered negligible, the dynamical nonlinearities may significantly contribute to the specific timing and phasing of feeding behaviors. Nonlinear properties have been characterized in a limited number of *Aplysia* muscles (Yu et al. 1999; Sukhnandan et al. 2024), and previous models indicate that they have important behavioral implications (Sutton et al. 2004b; Novakovic et al. 2006). Given the observed convergence to $0^{th}$ order dynamics in the biomechanics (Fig. 11), it may be possible to neglect the force-velocity properties of these muscles, modeling only their force-length properties and nonlinear activation dynamics in future model iterations.

#### Parameter sensitivities

Despite the use of linear spring-like mechanics and linear dynamics in the activation of the muscle, the overall dynamics of the system remain nonlinear due to the use of piecewise linear functions, rigid and discontinuous constraints, and the use of a discontinuous neural model. The nonlinearity in the dynamics makes it important to consider not just the specific parameters that are utilized but also the surrounding neighborhood in parameter space to understand the stability of the model. This stability is informative not just in its relationship to the numerical results and the robustness of the solutions. The interpretation of the local stability of a neuromechanical model and the changes in this stability throughout parameter space can provide important biological and control insights (Jackson et al. 2023; Wang et al. 2022a; Lobato-Rios et al. 2022; Tytell et al. 2010). The task of performing this analysis is made more difficult by the scale of the parameter space to investigate. For this model, nearly 50 parameters between the biomechanical and neural models needed to be estimated. Some of these could be independently calibrated using existing data (See Supporting Information Section 2 for details of the calibration of the muscle time constants), but roughly 80% of them could only be tuned to behavioral data. This was performed manually by the authors choosing model parameters, running simulations, and obtaining heuristic “sensitivities” by observing the changes in the model behavior. While performing a more systematic review of the parameter space will be important for this model, a full sensitivity analysis was considered beyond the scope of this work. The performance of this sensitivity analysis is also made more difficult by the choice of neural models because the discontinuous nature of the Boolean framework prevents the use of variational approaches that have been utilized in other neuromechanical analyses (Wang et al. 2022a, 2021), and therefore, it will be beneficial to use continuous dynamical neural models in future model iterations. Sensitivities around the hand-tuned parameters could be obtained approximately using numerical perturbation experiments. Alternatively, Monte Carlo sampling methods could be implemented to observe a larger subset of the parameter space to obtain information about global sensitivities (Jackson et al. 2023; Wang et al. 2022b).

#### Muscles are more than active line elements

While the model produces good quantitative matches to the animal data overall, there are still areas where improvement is needed to match the full cycle kinematics and neuromechanics better. First, it was observed that the rate of protraction was too fast: the slope of the odontophore translation curve is too high during protraction for swallowing and rejection behaviors. Although the range of motion in the behaviors was in good agreement with the animal data, the model produced this translation in a shorter amount of time than the animal, as shown in the higher Percent Protraction metric for the animals. Next, in both biting and rejection behaviors, the hinge was ineffective at producing retraction, and the model had to rely on the I3 to generate active force to compensate. While this produces kinematics that agree with the animal data, we know that in the animal, the I3 is not the sole retractor. In biting behaviors, while there is some activity in the I3 motor neurons, the frequency of firing is insufficient to generate large forces (Lu et al. 2015). The hinge produces forces during biting protraction that are sufficient to retract the odontophore (Sutton et al. 2004a). In rejections, the firing of B3/B6/B9 is delayed and the hinge muscle initiates retraction (Ye et al. 2006). Finally, in both behaviors for which we have animal data, the model grossly underestimates the rotational range of motion of the odontophore. This is because, while the odontophore is free to rotate inside the I3 lumen, there are no force-generating elements that have sufficient mechanical advantage to generate this rotation until the odontophore has exited the lumen and can effectively “fall” backward. These discrepancies all provide evidence against assumptions A2 and A3 and point to two major mechanical shortcomings in the model. First, in agreement with assumption A3, the model I3 currently generates no passive forces other than the inequality constraints that act to resist protraction. In reality, the odontophore has to push open and stretch the I3 muscle to protract. These passive forces would help to slow protraction and assist in retraction without requiring the I3 to generate force actively. The second shortcoming is the general inability of the hinge to produce significant force. Currently, in agreement with assumption A2, the hinge is modeled as a single line-element muscle, but because the ventral I1 can shorten as the odontophore enters the I3 lumen, the hinge stays very near its rest length throughout feeding. This means it can generate very little force and exhibits minimal mechanical advantage in the system. In contrast, in the animal, the hinge is not only stretched but is also bent as the odontophore rotates into the I3 lumen, acting akin to a beam structure. This bending generates a reaction torque, both working to resist protraction and to generate additional rotation while the odontophore is within the I3 lumen. Additionally, as the hinge contracts, the bending stiffness of the hinge will increase, subsequently increasing the restoring torque on the odontophore and further assisting retraction and rotation. By not including this bending/rotational stiffness in the hinge, the model insufficiently couples the translation and rotation of the odontophore, allowing the odontophore to slide along the long axis of the buccal mass with no muscular or passive structure applying significant rotations. These simplifying assumptions were made following a demand-driven complexity analysis of the system in attempts to improve the model’s anatomical accuracy while minimizing the increase in computational complexity. However, the results suggest that this simplification is inadequate to fully model the system, and in future versions of the model, the passive stiffness of the I3 lumen and the bending stiffness of the hinge should be incorporated.

#### Need for a shape-changing odontophore

Finally, one significant simplification in this model is the assumption that the odontophore has a constant shape. This simplification (or further reductions of the odontophore to a point mass) has been utilized in multiple previous models of the system (Sutton et al. 2004b; Kundu et al. 2022; Webster-Wood et al. 2020). During the demand-driven complexity analysis, this assumption was considered sufficient, and therefore, it was not worth the additional computational cost to incorporate a deformable grasper. However, in the animal, the odontophore changes shape throughout the feeding cycle across all three major axes (Neustadter et al. 2007). It has been hypothesized that this shape change influences the mechanical advantage of the different muscles in the buccal mass at different stages of feeding behavior (Sutton et al. 2004b; Novakovic et al. 2006). These hypotheses cannot be addressed in this model because of the fixed shape of the odontophore and lack of a length-tension model for the muscles (Yu et al. 1999).

The lack of a shape-changing odontophore also impacts the model observables and how they are compared to the animal data. This happens because there are additional internal degrees of freedom in the odontophore that are not captured in the model. Translation in the animal is measured as the distance from the jaw line to the anterior edge of the odontophore (Neustadter et al. 2007). In the model, this becomes simply an affine function of the center of mass position and head position. However, in the animal, as the odontophore deforms, the anterior edge may move, even if the center of mass does not. These deformation-based “translations” cannot be captured by the model. This mismatch due to the rigid odontophore is likely even greater for the measured rotation of the odontophore. In the model, the angle of the odontophore is measured from the vertical jaw line to a fixed axis in the rigid odontophore. As with translation, this becomes an affine function of the center of mass angle in the model. In the animal data, the radular stalk rotation is measured by a combined angle from the jaw line to I6 and from I6 to the radular stalk. As the animal feeds and the odontophore changes shape, both the anterior line of the I6 and the radular stalk can move within the odontophore. Thus, the measured angle of the jaw line to the radular stalk may change with no rigid body rotation of the odontophore. These changes cannot be fully captured in the model without similar internal degrees of freedom or ways to calculate appropriate analogs. For a more complete treatment of the system, a shape-changing odontophore should be incorporated in the future and is likely to significantly improve the match between animal data and the model predictions, particularly in rotation, which could justify the additional computational cost.

## Supplementary information

S1 Text. Computational methods for model observables, parameter estimation, statistical analysis, neural model equations, and mechanical variable definitions. In this supporting information, we provide additional details related to the calculation of model observables, the process of model parameter estimation, the statistical analysis performed in this paper, the equations governing the neural model, and the definitions of the variables and symbols in the mechanical model. S1 Text contains five figures and three tables. Fig. S1 shows the results of estimating the time constant of the I2 muscle based on the model presented in Yu et al. (1999). Fig. S2 presents model fits for the time constants of the I4, hinge, and anterior I3 muscles using data reported in Morton and Chiel (1993), Sutton et al. (2004a), and McManus et al. (2014), respectively. Fig. S3 shows the resulting bootstrapped distributions for the animal cycle durations for biting, unloaded swallowing, and loaded swallowing. Fig. S4 shows the resulting bootstrapped distributions for the Percent Protraction metric for the same behaviors. Finally, Fig. S5 reports the results of the computational power analysis performed on the Equivalence Test for the different available sample sizes. Tables in S1 Text provide the parameters used in the model, with Table 1 giving the mechanical model parameters, Table 2 the neural threshold parameters, and Table 3 additional (non-threshold) neural parameters. Finally, Table 4 contains a list of the symbols and variables used in the mechanical model, along with a brief description.

## Supplementary Information

Below is the link to the electronic supplementary material.

Supplementary file 1 (pdf 3162 KB)
